# Influence of receptor selectivity on benefits from SGLT2 inhibitors in patients with heart failure: a systematic review and head-to-head comparative efficacy network meta-analysis

**DOI:** 10.1007/s00392-021-01913-z

**Published:** 2021-09-08

**Authors:** Tobias Täger, Lutz Frankenstein, Dan Atar, Stefan Agewall, Norbert Frey, Morten Grundtvig, Andrew L. Clark, John G. F. Cleland, Hanna Fröhlich

**Affiliations:** 1 Department of Cardiology, Angiology, and Pneumology, University Hospital Heidelberg, Im Neuenheimer Feld 410, 69120 Heidelberg, Germany; 2 Department of Cardiology, Oslo University Hospital Ulleval and Institute of Clinical Sciences, University of Oslo, Oslo, Norway; 3 Medical Department, Innlandet Hospital Trust Division Lillehammer, Lillehammer, Norway; 4 Hull University Teaching Hospitals NHS Trust, Hull, UK; 5 National Heart & Lung Institute, Royal Brompton & Harefield Hospitals, Imperial College London, London, England; 6 Robertson Centre for Biostatistics and Clinical Trials, Glasgow, UK

**Keywords:** Heart failure, Diabetes mellitus, Sodium glucose cotransporter 2 inhibitor, Receptor selectivity, Outcome

## Abstract

**Background:**

Receptor selectivity of sodium-glucose cotransporter-2 inhibitors (SGLT2i) varies greatly between agents. The overall improvement of cardiovascular (CV) outcomes in heart failure (HF) patients varies between trials. We, therefore, evaluated the comparative efficacy of individual SGLT2i and the influence of their respective receptor selectivity thereon.

**Methods:**

We identified randomized controlled trials investigating the use of SGLT2i in patients with HF—either as the target cohort or as a subgroup of it. Comparators included placebo or any other active treatment. The primary endpoint was the composite of hospitalization for HF or CV death. Secondary outcomes included all-cause mortality, CV mortality, hospitalization for HF, worsening renal function (RF), and the composite of worsening RF or CV death. Evidence was synthesized using network meta-analysis. In addition, the impact of receptor selectivity on outcomes was analysed using meta-regression.

**Results:**

We identified 18,265 patients included in 22 trials. Compared to placebo, selective and non-selective SGLT2i improved fatal and non-fatal HF events. Head-to-head comparisons suggest superior efficacy with sotagliflozin as compared to dapagliflozin, empagliflozin or ertugliflozin. No significant difference was found between canagliflozin and sotagliflozin. Meta-regression analyses show a decreasing benefit on HF events with increasing receptor selectivity of SGLT2i. In contrast, receptor selectivity did not affect mortality and renal endpoints and no significant difference between individual SGLT2i was noted.

**Conclusion:**

Our data point towards a class-effect of SGLT2i on mortality and renal outcomes. However, non-selective SGLT2i such as sotagliflozin may be superior to highly selective SGLT2i in terms of HF outcomes.

**Supplementary Information:**

The online version contains supplementary material available at 10.1007/s00392-021-01913-z.

## Introduction

Sodium-glucose cotransporter-2 inhibitors (SGLT2i) had been established in type 2 diabetes (T2D) care for some time when their positive impact on cardiovascular (CV) outcomes—including heart failure (HF)—was recognized [1–4]. A potential superiority of SGLT2i over other hypoglycemic agents was suspected from observational databases [5] and in the following years, their prognostic value was extended to patients with HF with or without T2D for dapagliflozin in DAPA-HF [6], empagliflozin in EMPEROR-Reduced [7], and sotagliflozin in SOLOIST-WHF [8].

Receptor selectivity of SGLT2i varies greatly—while dapagliflozin and empagliflozin are selective SGLT2i, sotagliflozin is a non-selective inhibitor. Moreover, effects on individual CV endpoints differ between trials. It is thus unclear whether (a) the observed benefits represent a drug-specific rather than a class effect and (b) if the extent of the respective CV-benefit is modulated by the individual receptor selectivity. As there are no published prospective head-to-head comparisons of SGLT2i—and to the best of our knowledge none are planned, either—the comparative efficacy of individual SGLT2i in patients with HF remains to be elucidated.

To this end, the direct comparisons of the respective numeric values of effect-measures from the aforementioned trials (so-called naïve comparisons) are statistically inadequate. Standard (conventional) meta-analysis cannot address this question either because the baseline assumption of conventional meta-analysis is a class-effect as it essentially groups verum vs. placebo to calculate a summary effect measure. We, therefore, compared the CV benefits of selective and non-selective SGLT2i in patients with HF using a network meta-analysis (NMA).

## Methods

NMA is an extension of pairwise (conventional) meta-analysis in which multiple interventions are compared both directly within randomized controlled trials (RCTs) and indirectly, across trials, based on a common comparator. NMA has advantages over pair-wise meta-analysis, such as clarification of outcomes from multiple trials including several common comparators and indirect calculation of effects when direct comparisons between important treatments are not available. Also, NMA can provide increased statistical power and cross-validation of the observed treatment-effect of weak connections, given reasonable network connectivity and sufficient sample-sizes. This results in greater precision of treatment-effect estimates and the ability to rank all the interventions in a coherent way.

We performed the present review following the Preferred Reporting Items for Systematic Reviews and Meta-analyses (PRISMA) extension statement for reporting systematic reviews incorporating NMAs of health care interventions [9–12]. The protocol of the NMA was prospectively registered at PROSPERO (registration ID: CRD42020178502).

### Identification and selection of trials

We searched PubMed and www.clinicaltrials.gov up to November 24th 2020 for RCTs investigating the use of gliflozins in patients with HF. Both selective and non-selective SGLT2i were considered. Details of the search strategy are provided in the supplementary material. Two reviewers independently screened citations against the following predefined selection criteria.

*Study design* We included prospective RCTs investigating treatment with SGLT2i. There were no restrictions regarding the date of publication, language or sample size.

*Population* We selected trials including adults (≥ 18 years) with a history of HF or a diagnosis of prevalent, stable HF. Patients with HF with reduced, mid-range or preserved left ventricular ejection fraction (LVEF) were considered. There were no restrictions regarding sex, ethnic group, or dose of SGLT2i. Patients with and without diabetes were included. Trials that included solely patients with HF as well subgroups of patients with HF from RCTs with broader inclusion criteria were considered.

*Interventions* Treatment with either canagliflozin, dapagliflozin, empagliflozin, ertugliflozin, ipragliflozin, luseogliflozin, licogliflozin, remogliflozin, sergliflozin, sotagliflozin or tofogliflozin for at least 12 weeks. An arbitrary limit of 12 weeks was chosen to allow sufficient time for the RCT to accrue events.

*Comparators:* placebo or standard medical care.

*Outcomes:* the primary outcome was the composite of hospitalization for HF or CV death. The primary outcome was chosen since it has been reported in large SGLT2i trials and it is relevant to patients with HF. Secondary endpoints included all-cause mortality, CV mortality, hospitalization for HF, worsening renal function (RF), and the composite of worsening RF or CV death. Following the criteria used in trials, worsening RF was defined as doubling of serum creatinine, sustained 40–50% reduction in the estimated glomerular filtration rate (eGFR), end-stage renal disease, initiation of renal replacement therapy, or renal death.

### Data extraction and quality assessment

All relevant articles were independently reviewed by two investigators to assess the eligibility of the article and abstract with standardized data abstraction forms, and disagreement was resolved by a third investigator. For each trial included, details were extracted on trial design, patient characteristics, interventions, and outcomes. The quality of included trials was assessed using the Cochrane Collaboration Criteria [13].

### Statistical analyses

This NMA was conducted with Stata software 15.0 (StataCorp, College Station, TX, USA) using the network family of commands [14, 15]. A random effects model was applied. The NMA was performed to obtain estimates for outcomes of primary and secondary end-points, presented as odds ratio (OR) and 95% confidence intervals (CI) for binary outcomes. The plot of a network of drugs was used as a visual representation of the evidence-base and offered a concise description of its characteristics. It consists of nodes representing the interventions being compared and edges representing the available direct comparisons (comparisons evaluated in at least one trial) between pairs of interventions [15–17]. The quality of treatment effect estimates was rated following the Grading of Recommendations Assessment, Development and Evaluation (GRADE) approach [18, 19]. To make the rank of treatments, we used the surface under the cumulative ranking probabilities (SUCRA)—a transformation of the mean rank that accounts both for the location and the variance of all relative treatment-effects [20]. SUCRA values range from 0 to 1.0. The higher the SUCRA value, and the closer to 1.0, the higher the likelihood that a therapy is in the top rank or one of the top ranks; the closer to 0 the SUCRA value, the more likely that a therapy is in the bottom rank, or one of the bottom ranks [21]. To check for a publication bias, we designed a funnel plot [15]. Consistency of results was evaluated in each loop by calculation of an inconsistency factor and statistical significance determined via *z* test [17, 22].

We evaluated the influence of receptor selectivity on study outcome through meta-regression using the Stata metareg command on study-level summary data [23]. Meta-regression determines the extent to which statistical heterogeneity between the values of the respective effect measures of multiple studies can be related to one or more characteristics of the studies—receptor selectivity in our case. To test the stability of the results, we performed sensitivity analyses by restricting analyses to patients with a diagnosis of both HF and T2D. Data on different dosages of active treatments and/or comparators were pooled for each trial. Inter-rater agreement statistic (Kappa, 95% CI) was calculated according to Cohen [24]. All *p* values were two-tailed with the statistical significance arbitrarily set at < 0.05.

## Results

### Literature search

The search strategy yielded 24 eligible records reporting results from 22 trials [6–8, 25–45]. For two trials, results were not published in a journal article but they were extracted from www.clinicaltrials.gov (NCT03448406 and NCT 03485222). The flowchart of the trial selection process is shown in Online Fig. 1. Agreement between reviewers was excellent (*κ* = 0.833, 95% CI 0.677–0.990).

Ten trials investigated the use of SGLT2i in T2D and reported subgroup analyses from a total of 7084 patients with concomitant HF [25–29, 33, 34, 41]. Twelve trials including 11,181 patients prospectively selected patients with HF [6–8, 30–32, 35, 36, 38, 42–45], adding up to a total of 18,265 patients with HF included in the present NMA. All but one trial compared SGLT2i with placebo, and no trials directly compared two different SGLT2i.

Nine trials studied the use of dapagliflozin in a total of 7370 patients with HF. Three trials studied canagliflozin (*n* = 2149), eight trials studied empagliflozin (*n* = 5566) and one trial studied ertugliflozin in patients with HF (*n* = 1958). The non-selective SGLT2i licogliflozin and sotagliflozin were each studied in one trial (*n* = 124 and *n* = 1222, respectively). No trials were identified studying the use of ipragliflozin, luseogliflozin, remogliflozin, sergliflozin, or tofogliflozin in patients with HF. Pharmacokinetic characteristics of SGLT2i included in the present NMA are shown in Online Table 1. For characteristics of trials, please refer to Table 1.

**Table 1:** Baseline characteristics of included trials

Record	Trial*	NCT number	Year	Active treatment	Comparator	Centers (*n*)	Patients (*n*)	Patients with HF (*n*, %)	Duration (weeks)	FU in patients with HF (py)
Bhatt [8]	SOLOIST-WHF	NCT03521934	2020	Sotagliflozin	Placebo	306	1222	1222 (100)	39	916.5
Butler [25], Fitchett [26, 27]	EMPA-REG-OUTCOME	NCT01131676	2016–2019	Empagliflozin	Placebo	590	7020	706 (10.1)	164	1835.6
Cannon [28], Cosentino [29]	VERTIS CV	NCT01986881	2020	Ertugliflozin	Placebo	567	8238	1958	42	1581.5
Carbone [30]	CANA-HF	NCT02920918	2019	Canagliflozin	Sitagliptin	1	36	36 (100)	12	8.3
de Boer [31]	Licogliflozin vs. empagliflozin vs. placebo	NCT03152552	2020	Licogliflozin	Empagliflozin, Placebo	55	124 ^b^	124 (100)	12 ^b^	28.6^†^
Jensen [32]	EMPIRE-HF	NCT03198585	2020	Empagliflozin	Placebo	2	190	190 (100)	12	43.8
Kato [33]	DECLARE	NCT01730534	2019	Dapagliflozin	Placebo	882	17,160	1987 (11.6)	218.4	8345.4
Kosiborod [34] ^‡^	1. Moderate KD (56) 2. Add-on to sulfonylurea [57] 3. Add-on to insulin [58] 4. High CV risk [59] 5. High CV risk [60]	1. NCT00663260 2. NCT00680745 3. NCT00673231 4. NCT01031680 5. NCT01042977	2014 2011 2012 2015 2014	Dapagliflozin	Placebo	111 84 126 141 173	252 592 808 922 964	19 (7.5) 13 (2.2) 18 (14.3) 118 (12.8) 152 (15.8)	52 48 48 52 § 52 §	19 12 16.6 922 964
Lee [35]	SUGAR-DM-HF	NCT0348509	2020	Empagliflozin	Placebo	15	105	105 (100)	36	72.7
McMurray [6, 36], Petrie [37]	DAPA-HF	NCT03036124	2019	Dapagliflozin	Placebo	417	4744	4744 (100)	78.8	7195.1
Nassif [38]	DEFINE-HF	NCT 02653482	2019	Dapagliflozin	Placebo	26	263	263 (100)	12	60.7
Packer [7], Anker [39]	EMPEROR-Reduced	NCT03057977	2020	Empagliflozin	Placebo	520	3730	3730 (100)	69	4973.3
Perkovic [40]	CREDENCE	NCT02065791	2019	Canagliflozin	Placebo	690	4397	652 (14.8)	136	1708.2
Radholm [41]	CANVAS Program	NCT01032629 and NCT01989754	2018	Canagliflozin	Placebo	667	10,142	1461 (14.4)	188.2	5282.1
Santos-Gallego [42]	EMPA-TROPISM	NCT 03485222	2020	Empagliflozin	Placebo	1	84	84	26	42
Singh [43]	REFORM	NCT02397421	2020	Dapagliflozin	Placebo	1	56	56	52	56
Boehringer Ingelheim [44] ^∥^	EMPERIAL-Reduced	NCT03448419	2020	Empagliflozin	Placebo	109	312	312	12	72
Boehringer Ingelheim [45]^∥^	EMPERIAL-Preserved	NCT03448406	2020	Empagliflozin	Placebo	108	315	315	12	72.7

*HF* heart failure, *KD* kidney disease, *T2D* type 2 diabetes mellitus

*Acronym/short title

^†^Of the 124 patients randomized in the study, 80 were discontinued due to early study termination, with 44 patients completing the 12-weeks study

^‡^This record presents a HF subgroup meta-analysis from five randomized controlled trials. Patients with HF included in any of the five trials have been identified retrospectively and data have been pooled for joint analyses

^§^24-week trial plus 28-week (52-week) plus 52-week (104-week) extension period

^∥^At the time of the literature search, results have not been published in a journal but were extracted from www.clinicaltrials.gov

The network plots with respect to different endpoints are presented in Online Fig. 2.

### Patient characteristics

Patients were generally aged between 56 and 74 years and 22–44% were women. Mean LVEF was reported in nine trials and varied between 26% [38] and 45% [43]. Most patients had eGFR > 60 mL/min/1.73m^2^. More than 80% received treatment with angiotensin-converting enzyme inhibitors, angiotensin receptor blockers, or angiotensin receptor neprilysin inhibitors, and 70–97% were treated with beta-blockers. The proportion of patients with concomitant T2D was reported in all but two trials [44, 45] and varied between 0 and 100%, totaling 12,762 (66.6%) patients (Online Table 2).

### Risk of bias

The overall risk of bias was low to intermediate. Online Fig. 3 presents the individual items of the risk of bias assessment for each trial. Although all data were from RCTs, for ten trials it derived from subgroup analyses [25–29, 33, 34, 40, 41], which, by definition, is not truly randomized. However, eight trials reported baseline characteristics of HF subgroups with respect to trial treatment, all of which demonstrated a good balance of patient characteristics between treatment groups [25–27, 29, 34, 41].

The primary combined endpoint of hospitalization for HF or CV death was reported in nine trials including 16,034 patients. All-cause mortality could be retrieved for all but two trials (*n* = 15,655 patients), whereas CV mortality was reported in 13 trials (*n* = 14,584 patients). Data on hospitalization for HF were available for 16,757 patients included in 17 trials, and renal outcomes were reported in five trials (*n* = 10,897 patients). The composite endpoint of worsening RF or CV death was reported in three (*n* = 1391 patients) trials. There was no systematic association between type or size of the trial or the publication date and any pattern of missing endpoint information. The comparison-adjusted funnel plot for the primary endpoint was symmetrical, suggesting the absence of small-trial effects and publication bias (Online Fig. 4).

### Outcomes

#### Primary outcome

**Fig. 1:**
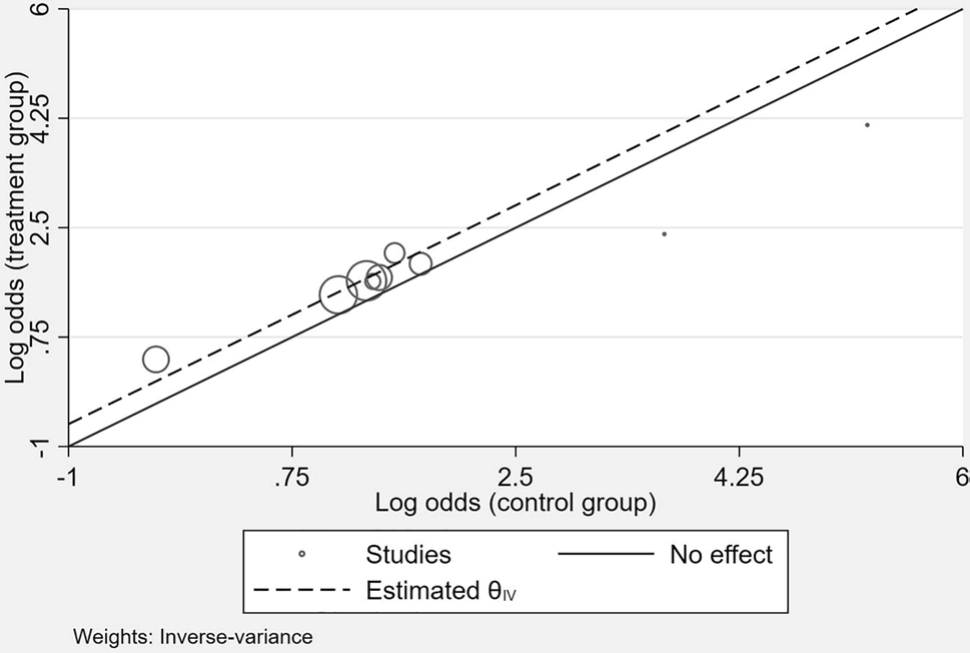
L’Abbé-plot of trials investigating the effects of SGLT2i on the composite outcome of hospitalizations for HF or CV death. *CV* cardiovascular, *HF* heart failure. The l’Abbé-plot plots the event rate in the experimental (intervention) group against the event rate in the control group. Trials in which the experimental treatment proves better than the control will be in the upper left of the plot, between the y axis and the line of equality. If experimental treatment is no better than control then the point will fall on the line of equality, and if control is better than experimental then the point will be in the lower right of the plot, between the *x* axis and the line of equality. The symbol size represents the sample size of the respective trials

As shown in Fig. 1, there was a consistent benefit on the composite of hospitalization for HF or CV death across different SGLT2i trials*.*

**Fig. 2:**
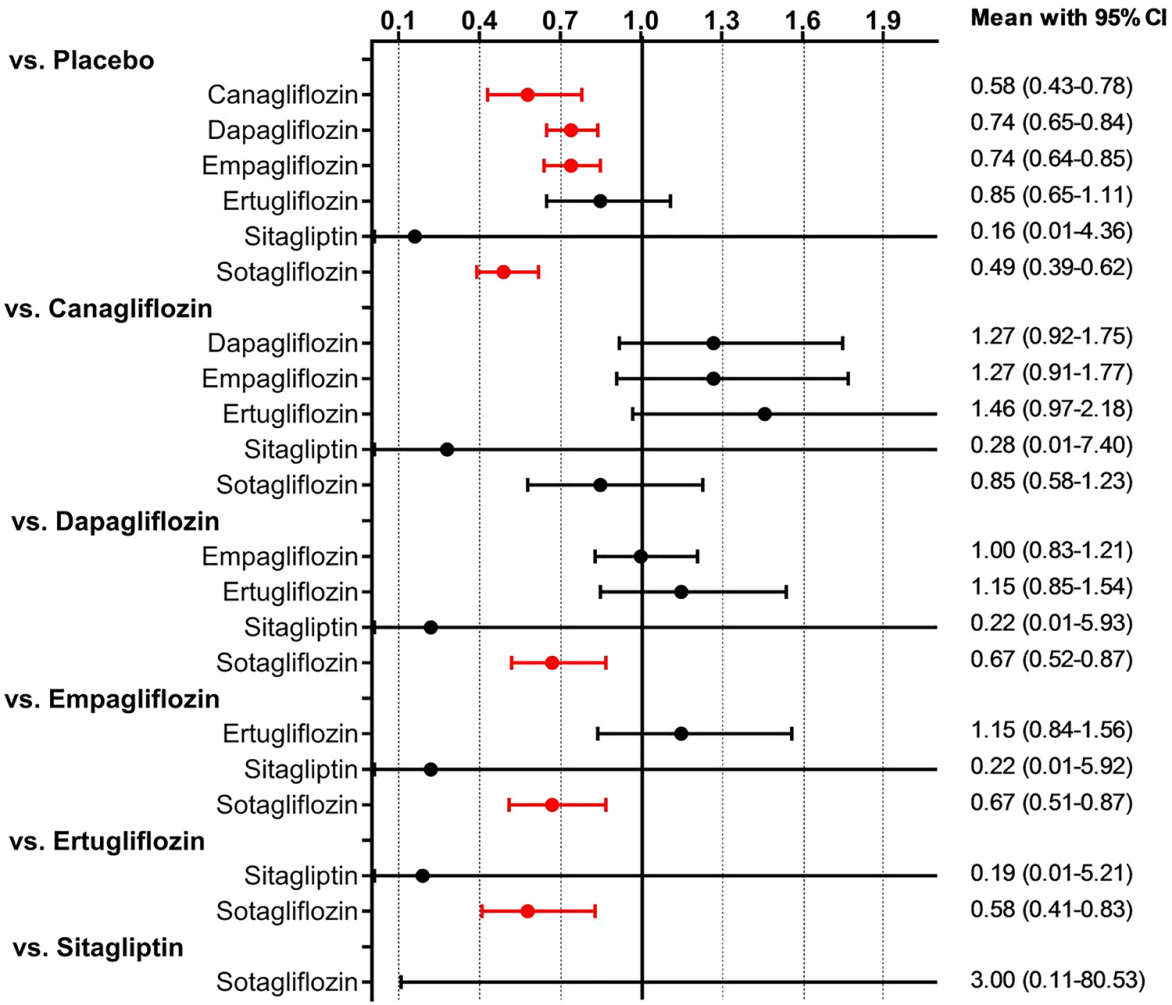
Predictive interval plot of individual SGLT2i for the combined primary endpoint of hospitalization for HF or CV death. *CV* cardiovascular, *HF* heart failure, *SGLT2i* sodium-glucose cotransporter 2 inhibitor. The predictive interval plot represents a forest plot of the joint estimated summary effects from both direct and indirect comparisons along with their confidence intervals. Significant summary effects are shown in red

**Fig. 3:**
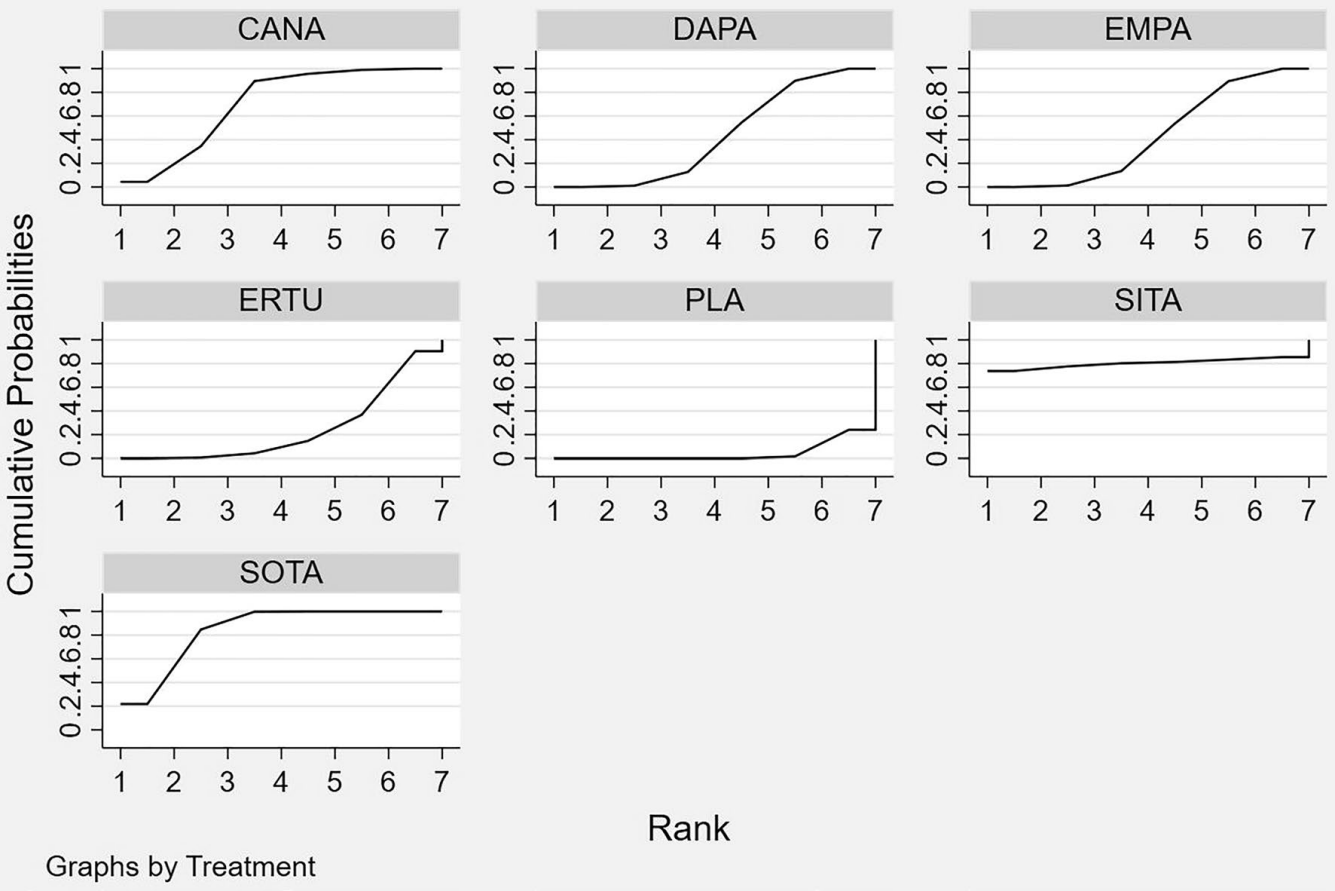
Graphical ranking of SGLT2i based on SUCRA values (hospitalization for HF or CV death). *CANA* canagliflozin, *CV* cardiovascular, *DAPA* dapagliflozin, *EMPA* empagliflozin, *ERTU* ertugliflozin, *HF* heart failure, *PLA* placebo, *SITA* sitagliptin, *SOTA* sotagliflozin

Network comparisons of individual SGLT2i included studies on the use of canagliflozin (*n* = 2; 1497 patients), dapagliflozin (*n* = 2; 6731 patients), empagliflozin (*n* = 3; 4626 patients), ertugliflozin (*n* = 1; 1958 patients) or sotagliflozin (*n* = 1; 1222 patients). No data were available for licogliflozin. The predictive interval plot summarizing the relative mean effects along with the impact of heterogeneity on the respective confidence interval (= the predictive interval) of each (network) comparison is shown in Fig. 2*.* When compared to placebo, canagliflozin, dapagliflozin, empagliflozin, and sotagliflozin reduced the composite endpoint of hospitalizations for HF or CV death significantly. A non-significant benefit was found with ertugliflozin. Indirect head-to-head comparisons showed a significant benefit of sotagliflozin over dapagliflozin, empagliflozin, and ertugliflozin. In addition, our results visually suggest a benefit of canagliflozin over dapagliflozin, empagliflozin, and ertugliflozin. However, confidence intervals just cross the line of null effect. No significant difference was found between canagliflozin and sotagliflozin. SUCRA values are presented in Table 2. The graphical display of the ranking based on the SUCRA values is shown in Fig. 3. No closed loops were formed and consequently, no inconsistency could be derived.

**Table 2:** Surface under the cumulative ranking curve (SUCRA) values for all endpoints

SUCRA	Hospitalization for HF or CV death	All-cause mortality	CV mortality	Hospitalization for HF	Worsening RF	Worsening RF or CV death
Canagliflozin	0.708	0.691	0.726	0.752	0.565	0.412
Dapagliflozin	0.432	0.611	0.608	0.337	0.442	n.a.
Empagliflozin	0.425	0.386	0.503	0.347	0.672	0.439
Ertugliflozin	0.243	n.a.	n.a.	0.473	n.a.	n.a.
Licogliflozin	n.a.	0.559	0.347	n.a.	n.a.	n.a.
Placebo	0.041	0.192	0.251	0.020	0.082	0.403
Sitagliptin	0.809	0.558	0.550	0.819	0.739	0.671
Sotagliflozin	0.842	0.504	0.515	0.751	n.a.	n.a.

SUCRA is a transformation of the mean rank that accounts both for the location and the variance of all relative treatment effects. The larger the SUCRA value, the better the rank of the treatment [20]

*CV* cardiovascular, *HF* heart failure, *n.a.* not available, *RF* renal function

**Fig. 4:**
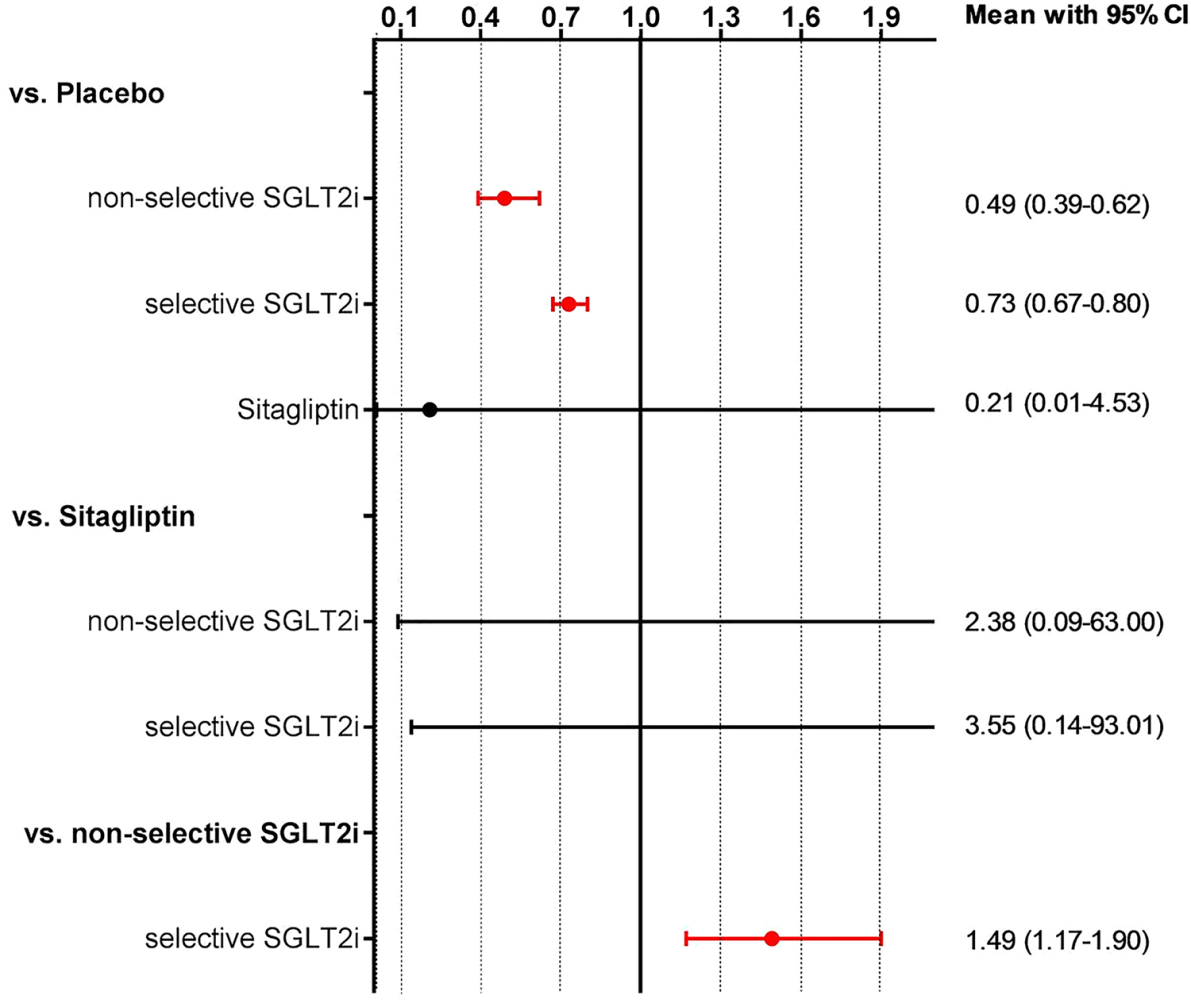
Predictive interval plot of selective vs. non-selective SGLT2i for the combined primary endpoint of hospitalization for HF or CV death. *CV* cardiovascular, *HF* heart failure, *SGLT2i* sodium-glucose cotransporter 2 inhibitor. The predictive interval plot represents a forest plot of the joint estimated summary effects from both direct and indirect comparisons along with their confidence intervals. Significant summary effects are shown in red

**Fig. 5:**
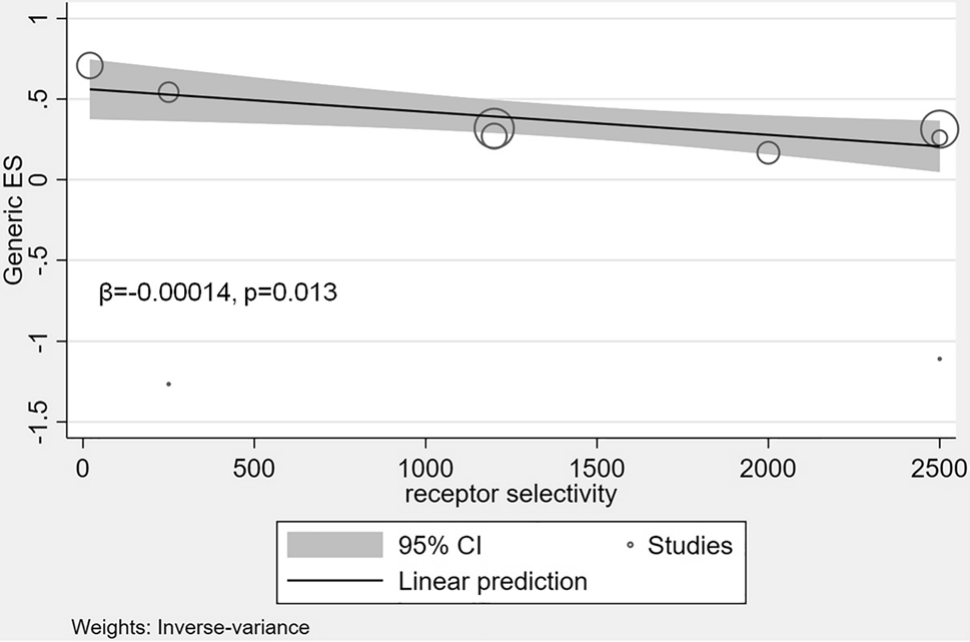
Relationship between effect size and receptor selectivity of SGLT2i for the combined primary endpoint of hospitalizations for HF or CV death. *CI* confidence interval, *CV* cardiovascular, *ES* effect size, *HF* heart failure, *SGLT2i* sodium-glucose cotransporter 2 inhibitor. Each bubble represents an SGLT2i trial. The symbol size represents the sample size of the respective trials

In NMA of selective vs. non-selective SGLT2i, we found that both classes of SGLT2i improve the composite of hospitalization for HF or CV death when compared to placebo. However, indirect head-to-head-comparisons show a significant 49% benefit with non-selective SGLT2i as compared to selective SGLT2i (Fig. 4). Meta-regression analyses revealed a decreasing benefit on the primary outcome with increasing selectivity of SGLT2i (Fig. 5).

#### Secondary outcomes

##### All-cause mortality

Comparisons of individual SGLT2i included studies on the use of canagliflozin (*n* = 2; 1497 patients), dapagliflozin (*n* = 9; 7370 patients), empagliflozin (*n* = 8; 5565 patients), licogliflozin (*n* = 1; 124 patients), and sotagliflozin (*n* = 1; 1222 patients). No data were available for ertugliflozin. Although the effect estimates suggest that all SGLT2i improve survival when compared to placebo, the reduction in all-cause mortality was statistically significant only for dapagliflozin (Online Fig. 5). Indirect head-to-head comparisons, however, found no significant differences in survival between agents. The respective SUCRA values are presented in Table 2. The graphical display of the ranking based on the SUCRA values is shown in Online Fig. 6. Inconsistency could only be derived for one closed loop (empagliflozin—licogliflozin—placebo) and did not reach statistical significance [IF 0.95, 95% confidence interval (CI) 0.00–4.92].

NMA of SGLT2i classes found a 16% mortality reduction when compared to placebo, with no significant difference between selective and non-selective SGLT2i (Online Fig. 7). Accordingly, meta-regression analyses showed no relationship between effects on mortality and receptor selectivity of SGLT2i (Online Fig. 8).

##### CV mortality

Comparisons of individual SGLT2i included studies on the use of canagliflozin (*n* = 2; 1497 patients), dapagliflozin (*n* = 4; 7050 patients), empagliflozin (*n* = 6; 5039 patients), licogliflozin (*n* = 1; 124 patients), and sotagliflozin (*n* = 1; 1222 patients). No data were available for ertugliflozin. The results for CV mortality mirror those for all-cause mortality: When compared to placebo, the reduction in CV mortality was statistically significant only for dapagliflozin. Again, no significant differences in CV mortality were noted between individual SGLT2i (Online Fig. 9). SUCRA values are presented in Table 2. The graphical display of the ranking based on the SUCRA values is shown in Online Fig. 10. Inconsistency could only be derived for one closed loop (empagliflozin—licogliflozin—placebo) and did not reach statistical significance (IF 0.21, 95% CI 0.00–4.78). There was no relationship between CV mortality and receptor selectivity of SGLT2i (Online Figs. 11 and 12).

##### Hospitalization for HF

Comparisons of individual SGLT2i included studies on the use of canagliflozin (*n* = 2; 1497 patients), dapagliflozin (*n* = 9; 7370 patients), empagliflozin (*n* = 4; 4710 patients), ertugliflozin (*n* = 1; 1958 patients) or sotagliflozin (*n* = 1; 1222 patients). No data were available for licogliflozin. The results for hospitalization for HF mirror those for the combined outcome of hospitalization for HF or CV death: As shown in Online Fig. 13, all SGLT2i reduced hospitalizations for HF when compared to placebo, with sotagliflozin being superior to dapagliflozin and empagliflozin. The respective SUCRA values are presented in Table 2. The graphical display of the ranking based on the SUCRA values is shown in Online Fig. 14. No closed loops were formed and consequently, no inconsistency could be derived. NMA of selective vs. non-selective SGLT2i shows that both classes of SGLT2i improve hospitalizations for HF when compared to placebo, with a significant 36% benefit of non-selective SGLT2i over selective SGLT2i (Online Fig. 15). Again, the effect on HF events decreased with increasing receptor selectivity of SGLT2i (Online Fig. 16).

##### Worsening RF

Comparisons of individuals included studies on the use of canagliflozin (*n* = 2; 1497 patients), dapagliflozin (*n* = 2; 5007 patients) or empagliflozin (*n* = 2; 4429 patients). No data were available for ertugliflozin, licogliflozin or sotagliflozin. As displayed in the predictive interval plot (Online Fig. 17), canagliflozin, dapagliflozin and empagliflozin reduced worsening RF when compared to placebo with only empagliflozin reaching statistical significance. Indirect head-to-head comparisons found no significant differences in renal outcomes between individual SGLT2i. SUCRA values are presented in Table 2. The graphical display of the ranking based on the SUCRA values is shown in Online Fig. 18. No closed loops were formed and consequently, no inconsistency could be derived. Due to missing data, NMA of selective vs. non-selective SGLT2i were not possible. Meta-regression analyses showed no significant relationship between receptor selectivity and worsening RF in patients with HF (Online Fig. 19). However, due to the limited number of studies included in the analysis, confidence intervals are wide and results need to be interpreted with caution.

##### Worsening RF or CV death

Only three trials on the use of canagliflozin (*n* = 2; 688 patients) or empagliflozin (*n* = 1; 699 patients) in patients with HF reported the composite outcome of worsening RF and CV death [25, 30, 40]. Therefore, NMA calculations using a random effects model were not possible. The following results were obtained using a fixed-effects model and should be interpreted with caution. The predictive interval plot (Online Fig. 20) showed no significant effect of canagliflozin and empagliflozin on the composite endpoint of worsening RF or CV death when compared to placebo. There were no significant differences in the composite outcome between individual SGLT2i. SUCRA values are presented in Table 2. No closed loops were formed and consequently, no inconsistency could be derived. Due to missing data, NMA of selective vs. non-selective SGLT2i, as well as meta-regression analyses, were not possible.

For all endpoints including the respective outcome numbers per trial arm please also refer to Online Table 3.

#### Sensitivity analyses

Sensitivity analyses restricting analyses to patients with concomitant HF and T2D essentially confirmed our main results. However, confidence intervals of effect estimates with respect to HF or renal endpoints were wide and results thus need to be interpreted with caution. Detailed results from sensitivity analyses are presented in the supplementary material (supplemental results, Online Figs. 21–30).

## Discussion

Currently, no data exists on either the impact of receptor selectivity on the magnitude of risk reduction or the comparative CV effectiveness of individual SGLT2i—neither in patients with HF nor T2D. Using data from 18,265 patients enrolled in 22 trials, we found that all SGLT2i improve fatal and non-fatal HF events when compared to placebo. However, the benefits of SGLT2i increase with decreasing receptor selectivity, with sotagliflozin showing superior efficacy on HF outcomes. In contrast, receptor selectivity does not affect mortality or renal endpoints and no significant difference between individual SGLT2i was noted. It is here that our NMA significantly supplements and extents existing, conventional meta-analyses [46, 47] as it includes substantially more trials and patients and it provides statistical evidence for their underlying assumption—a class-effect—via NMA as the most appropriate tool.

To date, the mechanisms behind the benefits of SGLT2i in HF are not fully understood. Considering that SGLT2 receptors are not expressed in the human myocardium [48], it is difficult to reconcile whether and how SGLT2 inhibition could have direct effects on ventricular function [49]. In a randomized study on 56 patients with HF with reduced ejection fraction (HFrEF) and T2D, dapagliflozin did not have a significant effect on left ventricular remodelling as determined by cardiac MRI [43]. In addition, empagliflozin did not change NTproBNP levels in patients with mildly symptomatic HFrEF included in EMPIRE-HF [32]. Some authors have postulated that SGLT2i-mediated cardioprotective effects are secondary to indirect effects on sodium and calcium entry into cardiomyocyte cellular compartments [49]. Others propose that SGLT2i improve the efficiency of myocardial energetics by inducing a favourable shift in glucose and fat metabolism towards increased ketone substrate use [50–52]. Improved endothelial function and reduced vascular stiffening as well as increased diuresis and erythropoietin production may further contribute to the CV benefits seen with SGLT2i [49]. The combined inhibition of SGLT1 and SGLT2 is hypothesized to enhance the effects on renal sodium and glucose handling further via inhibition of both cotransporter subtypes in the proximal renal tubule [31, 53]. SGLT1 on the other hand has an important role in glucose absorption in the intestines—and in contrast to SGLT2, SGLT1 receptors are specifically expressed in the human myocardium [54, 55]. Left ventricular SGLT1 appeared upregulated in patients with HF in 71 patients with end-stage HF [56]. Here, ventricular myocardial SGLT1 expression correlated significantly with measures of cardiac remodelling and systolic function [56]. Whether and how the cardiac expression of SGLT1 contributes to the pronounced benefits seen with sotagliflozin in patients with HF needs to be elucidated. With respect to the dual SGLTi licogliflozin, investigations on the effects on HF events in patients with a prior diagnosis of HF are no longer pursued by the pharmaceutical developer and the company is focussing further research on patients with hepatic steatosis.

While results for HF-endpoints were robust, results for all-cause mortality were largely driven by DAPA-HF, which is the largest trial to date (*n* = 4744) that included solely patients with HFrEF with or without T2D [6]. In DAPA-HF, all-cause mortality was reduced by 17% and CV death by 18% with dapagliflozin. In contrast, the EMPEROR-Reduced trial reported a non-significant 8% reduction in all-cause and CV death with empagliflozin in patients with symptomatic HFrEF [7]. The recently published SOLOIST-WHF trial found a 18% reduction in deaths from any causes and a 16% reduction in CV death in patients with T2D who had recently been hospitalized for worsening HF [8]. However, due to the relatively small number of patients included in the trial (*n* = 1222), confidence intervals were wide and results did not reach statistical significance. To date, there are no prospective CV outcome trials with canagliflozin, ertugliflozin or licogliflozin in patients with HF. In large-scale trials involving patients with T2D, the risk reductions in CV death among patients with HF at baseline were 28% for canagliflozin, 45% for dapagliflozin, and 29% for empagliflozin, respectively [27, 33, 41]. In our study, indirect head-to-head comparisons including 15,655 patients from 20 trials revealed no significant differences in mortality reduction between individual SGLT2i. In addition, there was no difference between selective and non-selective SGLT2i with respect to all-cause or CV mortality. The different effects on mortality in DAPA-HF, EMPEROR-Reduced, and SOLOIST-WHF may thus be explained by differences in trial sizes and populations, as indicated by a significantly higher event rate in EMPEROR-Reduced and SOLOIST-WHF as compared to DAPA-HF [6–8].

For the prevention of worsening RF, the mean effect estimates in our analysis point towards a benefit with canagliflozin, dapagliflozin and empagliflozin compared to placebo, with only empagliflozin reaching statistical significance. No data were available for ertugliflozin, licogliflozin or sotagliflozin. Our results are largely driven by the EMPEROR-Reduced trial that observed a lower risk of the composite renal outcome in the empagliflozin group than in the placebo group [7]. In contrast, DAPA-HF reported a significantly greater increase in serum creatinine at eight months in those assigned to dapagliflozin compared to placebo [6]. Then again, the number of adverse renal events was similar between treatment groups [6]. In the CREDENCE trial, canagliflozin improved the composite primary endpoint of worsening RF or CV death in patients with T2D [40]. However, the number of events in the subgroup of patients with T2D and HF were similar between treatment groups (52 events with canagliflozin vs. 53 events with placebo). Again, we found no relationship between selectivity for SGLT2 and renal outcomes. However, the number of trials and patients in analyses was limited and should therefore be interpreted with caution. Further research is needed to clarify the effects of SGLT2i and the role of receptor selectivity on renal function in patients with HF.

Our study has several potential limitations. *First*, data for ertugliflozin, licogliflozin and sotagliflozin each stem from only one trial and are therefore susceptible to bias. Sotagliflozin is the only non-selective SGLT2i reporting data on HF outcomes and to the best of our knowledge, no other non-selective SGLT2i are currently being studied in patients with HF. The number of patients included in SOLOIST-WHF was rather small (*n* = 1222) and follow-up was limited to nine months due to loss of funding from the sponsor. In addition, SOLOIST-WHF was the only trial that enrolled patients admitted for acute HF, whereas other trials included patients with chronic stable HF. The selection of acutely decompensated HF patients, as well as the early termination of the trial, may have exaggerated the treatment effects of sotagliflozin. Results should therefore be interpreted with caution until more evidence on the use of sotagliflozin (or other non-selective SGTL2i) in patients with HF is available. *Second*, we identified twelve trials that solely included patients with HF, whereas ten trials were trials of T2DM that reported results for HF subgroups. By definition, randomization is invalid in subgroup analyses, thereby increasing the risk of bias. However, eight of these trials reported baseline characteristics of HF subgroups with respect to trial treatment, which demonstrated a good balance between treatment groups [25–27, 29, 34, 41]. Most importantly, background HF treatment was similar amongst prospective SGLT2i trials of HF and subgroup analyses of HF in SGLT2i trials of T2DM, with > 80% of patients receiving renin–angiotensin–aldosterone-system inhibitors and beta-blockers. *Third*, LVEF was reported in only nine trials, of which all but one enrolled patients with HFrEF. None of the T2D CV outcome trials reported LVEF. It is currently uncertain whether the effects of SGLT2 on HF outcomes differ for patients with reduced versus preserved LVEF (HFpEF). Recently, the trial sponsors Boehringer Ingelheim and Eli Lilly and Company announced that empagliflozin failed to improve exercise capacity as measured by 6 min walking distance in the EMPERIAL trials. EMPERIAL consisted of two phase III randomized, double-blind trials that included patients with HFrEF (EMPERIAL-reduced) or HFpEF (EMPERIAL-preserved) with our without diabetes. The number of patients included, however, was modest (312 in EMPERIAL-reduced and 315 in EMPERIAL-preserved), and follow-up was only 12 weeks. Then again, a subgroup analysis of the DECLARE-TIMI 58 trial showed that dapagliflozin improved CV outcomes in patients with HFrEF but not in those with HFpEF at baseline [33]. On-going trials such as the EMPEROR-preserved trial with empagliflozin and the DELIVER trial with dapagliflozin will help to clarify the role of SGLT2i in HFpEF.

## Conclusion

In conclusion, our data point towards a class-effect of SGLT2i on mortality and renal outcomes. However, non-selective SGLT2i such as sotagliflozin may be superior to highly selective SGLT2i in terms of HF outcomes. More studies are warranted to clarify the role of receptor selectivity of SGLT2i in the treatment of patients with HF.

## Supplementary Information

Below is the link to the electronic supplementary material.

Supplementary file1 (PDF 4039 KB)

## Data Availability

The present study is a network meta-analysis of published randomized trials. All data used for analyses are presented in the manuscript.

## Abbreviations

CV: Cardiovascular
HF: Heart failure
HFpEF: Heart failure with preserved ejection fraction
HFrEF: Heart failure with reduced ejection fraction
LVEF: Left ventricular ejection fraction
NMA: Network meta-analysis
RCT: Randomized controlled trial
SGLT2i: Sodium-glucose cotransporter 2 inhibitor
SUCRA: Surface under the cumulative ranking probabilities
T2D: Type 2 diabetes mellitus

## Code availability

Not available.
