# Supplementary material for: Influence of receptor selectivity on benefits from SGLT2 inhibitors in patients with heart failure: a systematic review and head-to-head comparative efficacy network meta-analysis
Source: Clin Res Cardiol. 2021 Sep 8;111(4):428–39. doi: 10.1007/s00392-021-01913-z (PMC8971161; doi:10.1007/s00392-021-01913-z)
Supplement: Supplementary file 1 — Supplementary file1 (PDF 4039 KB) [file 392_2021_1913_MOESM1_ESM.pdf]

SUPPLEMENTARY MATERIAL

Supplementary material to Tobias Täger*[1], MD; Lutz Frankenstein*[1], Prof.; Dan Atar[2], Prof.; Stefan Agewall[2], Prof.; Norbert Frey[1], Prof.; Morten Grundtvig[3], Prof.; Andrew L. Clark[4], Prof.; John G. F. Cleland[5], Prof.; Hanna Fröhlich[1], MD: Influence of receptor selectivity on benefits from SGLT2 inhibitors in patients with heart failure: a systematic review and head-to-head comparative efficacy network meta-analysis. Clinical Research in Cardiology 2021.

Corresponding author: Lutz Frankenstein, lutz.frankenstein@med.uni-heidelberg.de

CONTENT

SEARCH ALGORITHMS 4
Pubmed: 4
www.clinicaltrials.gov: 4
SUPPLEMENTARY RESULTS 4
Sensitivity analyses 4
Hospitalization for HF or CV death 4
All-cause mortality 5
CV mortality 5
Hospitalization for HF 5
Worsening RF 5
Worsening RF or CV death 5
SUPPLEMENTARY FIGURES 6
Online Figure 1: Flow chart of trial identification and selection 6
Online Figure 2: Network plots with respect to a) hospitalisation for HF or CV death, b) all-cause mortality, c) cardiovascular mortality, d) hospitalisation for HF, e) worsening RF, , and f) worsening RF or CV death. 7
Online Figure 3: Individual risk of bias assessment 11
Online Figure 4: Comparison funnel plot for the combined endpoint of hospitalisation for HF or CV death 12
Online Figure 5: Predictive interval plot of individual SGLT2i for hospitalisation all-cause mortality 13
Online Figure 6: Graphical ranking of SGLT2i based on SUCRA values (all-cause mortality) 14
Online Figure 7: Predictive interval plot of SGLT2i classes for all-cause mortality 15
Online Figure 8: Relationship between effect size and receptor selectivity of SGLT2i for all-cause mortality 16
Online Figure 9: Predictive interval plot of individual SGLT2i classes for CV mortality 17
Online Figure 10: Graphical ranking of SGLT2i based on SUCRA values (CV mortality) 18
Online Figure 11: Predictive interval plot of SGLT2i classes for CV mortality 19
Online Figure 12: Relationship between effect size and receptor selectivity of SGLT2i for CV mortality . 20
Online Figure 13: Predictive interval plot of individual SGLT2i for hospitalisation for HF 21
Online Figure 14: Graphical ranking of SGLT2i based on SUCRA values (hospitalisation for HF) 22
Online Figure 15: Predictive interval plot of SGLT2i classes for hospitalisations for HF 23
Online Figure 16: Relationship between effect size and receptor selectivity of SGLT2i for hospitalisations for HF 24
Online Figure 17: Predictive interval plot for worsening RF 25
Online Figure 18: Graphical ranking of SGLT2i based on SUCRA values (worsening RF) 26
Online Figure 19: Relationship between effect size and receptor selectivity of SGLT2i for worsening RF 27
Online Figure 20: Predictive interval plot for worsening RF or CV death 28

Online Figure 21: Predictive interval plot for hospitalisation for HF or CV death in subgroups of patients with both HF and T2D (sensitivity analysis) 29
Online Figure 22: Predictive interval plot for SGLT2i classes with respect to hospitalisation for HF or CV death in subgroups of patients with both HF and T2D (sensitivity analysis) 30
Online Figure 23: Predictive interval plot for all-cause mortality in subgroups of patients with both HF and T2D (sensitivity analysis) 31
Online Figure 24: Predictive interval plot for SGLT2i classes with respect to all-cause mortality in subgroups of patients with both HF and T2D (sensitivity analysis) 32
Online Figure 25: Predictive interval plot for CV mortality in subgroups of patients with both HF and T2D (sensitivity analysis) 33
Online Figure 26: Predictive interval plot for SGLT2i classes with respect to CV mortality in subgroups of patients with both HF and T2D (sensitivity analysis) 34
Online Figure 27: Predictive interval plot for hospitalisation for HF in subgroups of patients with both HF and T2D (sensitivity analysis) 35
Online Figure 28: Predictive interval plot for SGLT2i classes with respect to hospitalisations for HF in subgroups of patients with both HF and T2D (sensitivity analysis) 36
Online Figure 29: Predictive interval plot for worsening RF in subgroups of patients with both HF and T2D (sensitivity analysis) 37
Online Figure 30: Predictive interval plot for worsening RF or CV death in subgroups of patients with both HF and T2D (sensitivity analysis) 38
SUPPLEMENTARY TABLES 39
Online Table 1: Characteristics of SGLT2i included in analyses 39
Online Table 2: Characteristics of HF patients included in SGLT2i trials 41
Online Table 3: Endpoint characteristics of trials analysing the effects of SGLT2i in patients with HF 48

SEARCH ALGORITHMS

Pubmed:

heart failure AND ("SGLT2 inhibitor*" OR "SGLT2i" OR "Empagliflozin" OR "Dapagliflozin" OR "Canagliflozin" OR "Ertugliflozin" OR “Ipragliflozin” OR “Luseogliflozin” OR “Licogliflozin” OR “Remogliflozin” OR “Sergliflozin” OR “Sotagliflozin” OR “Tofogliflozin”) AND ("cardiovascular outcome" OR "cardiovascular death" OR "cardiovascular mortality" OR "all-cause mortality" OR "all-cause death" OR "death" OR "heart failure" OR "worsening renal function" OR "renal outcome" OR "renal composite") AND (trial OR random* OR controlled) NOT review

www.clinicaltrials.gov:

Condition or disease: heart failure

Other terms: Empagliflozin OR Canagliflozin OR Dapagliflozin OR Ertugliflozin OR Ipragliflozin OR Luseogliflozin OR Licogliflozin OR Remogliflozin OR Sergliflozin OR Sotagliflozin OR Tofogliflozin

Recruitment: terminated, completed

Age: adult (18–64), older adult (65+)

Sex: all

Study type: interventional

SUPPLEMENTARY RESULTS

Sensitivity analyses

To test the stability of the results, we performed sensitivity analyses by repeating calculations in the subgroup of patients with a diagnosis of both HF and T2D.

Hospitalization for HF or CV death

Data were available from eight trials including 11,365 patients with HF and T2D (7, 8, 27, 28, 30, 33, 37, 41, 62). A non-significant benefit with canagliflozin or sotagliflozin as compared to other SLT2i was noted. Accordingly, there was a non-significant benefit with non-selective over selective SGLT2i. However, confidence intervals are wide and results need to be interpreted with caution. The respective interval plots are shown in **Online Figure 21** and **Online Figure 22**.

All-cause mortality

Data were available from 13 trials including 8,051 patients with HF and T2D (8, 27, 30, 31, 32, 34, 37, 41, 43). When compared to placebo, dapagliflozin improved survival. However, no significant differences in all-cause mortality were noted between individual SGLT2i. In addition, no difference in mortality reduction was noted between selective and non-selective SGLT2i. The respective interval plots are shown in **Online Figure 23** and Online Figure 24**:** Predictive interval plot for SGLT2i classes with respect to all-cause mortality in subgroups of patients with both HF and T2D (sensitivity analysis) .

CV mortality

Data were available from nine trials including 9,526 patients with HF and T2D (8, 26, 30, 31, 33, 37, 39, 41, 43). No significant differences in CV mortality were noted between individual SGLT2i or SGLT2i classes. The respective interval plots are shown in **Online Figure 25** and **Online Figure 26**.

Hospitalization for HF

Data were available from 14 trials including 11,795 patients with HF and T2D (8, 27, 28, 30, 33, 34, 37-39, 41, 43, 62). A non-significant benefit with canagliflozin or sotagliflozin as compared to other SLT2i was noted. Accordingly, there was a non-significant benefit with non-selective over selective SGLT2i. However, confidence intervals are wide and results need to be interpreted with caution. The respective interval plots are shown in **Online Figure 27** and **Online Figure 28**.

Worsening RF

Data were available from four trials including 4,335 patients with HF and T2D (25, 30, 37, 41). No significant differences in worsening RF were noted between individual SGLT2i. The respective interval plot is shown in **Online Figure 29**. Due to missing data, comparisons of selective vs. non-selective SGLT2i were not possible.

Worsening RF or CV death

Data were available from three trials including 1,387 patients with HF and T2D (25, 30, 40). No significant differences in worsening RF or CV death were noted between individual SGLT2i. The respective interval plot is shown in **Online Figure 30**. Due to the low number of patients included in the analysis, however, results should be interpreted with caution. In addition, comparisons of selective vs. non-selective SGLT2i were not possible due to missing data.

SUPPLEMENTARY FIGURES

Online Figure 1: Flow chart of trial identification and selection

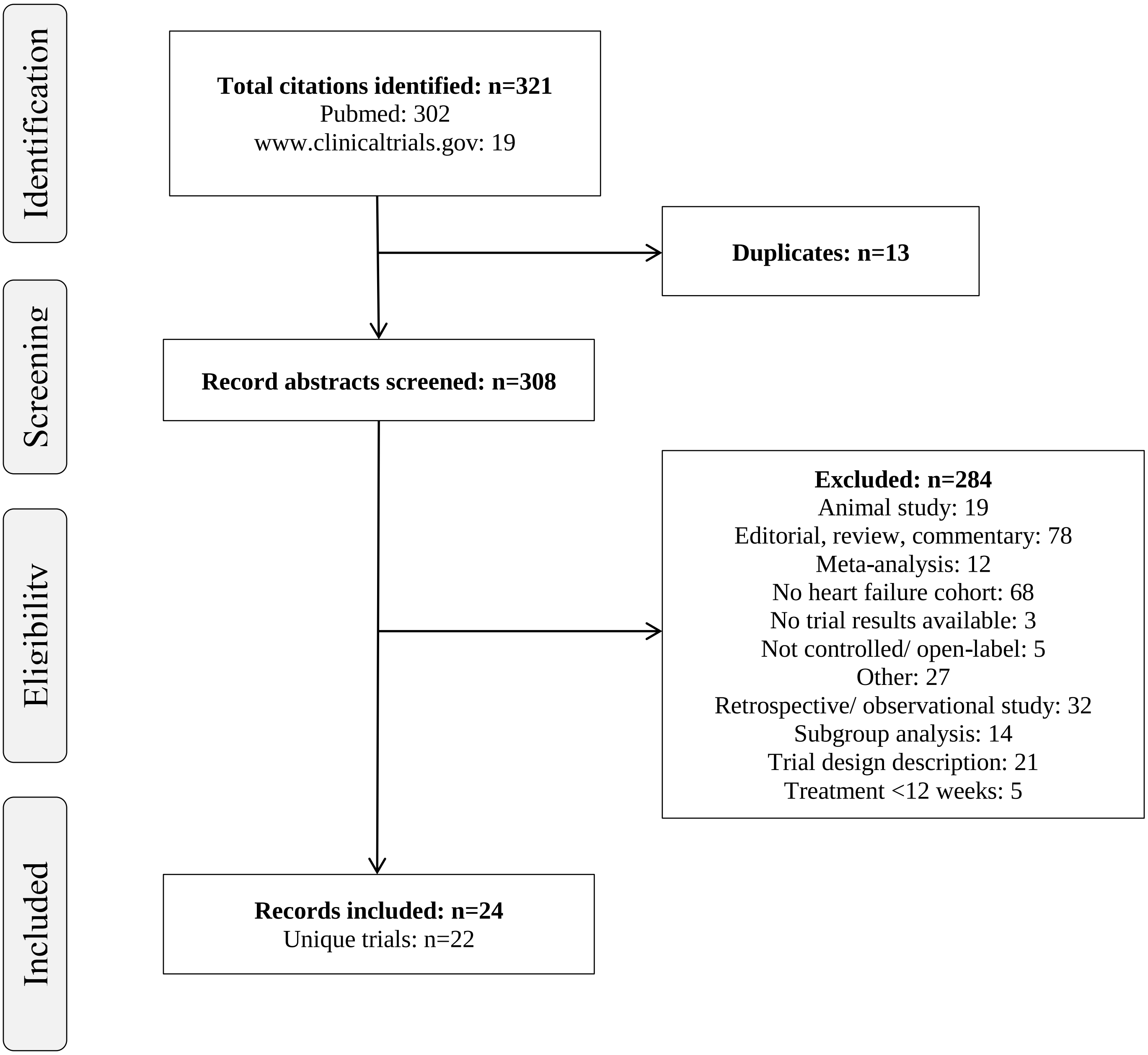

Online Figure 2: Network plots with respect to a) hospitalization for HF or CV death, b) all-cause mortality, c) cardiovascular mortality, d) hospitalization for HF, e) worsening RF, and f) worsening RF or CV death.

a) hospitalization for HF or CV death

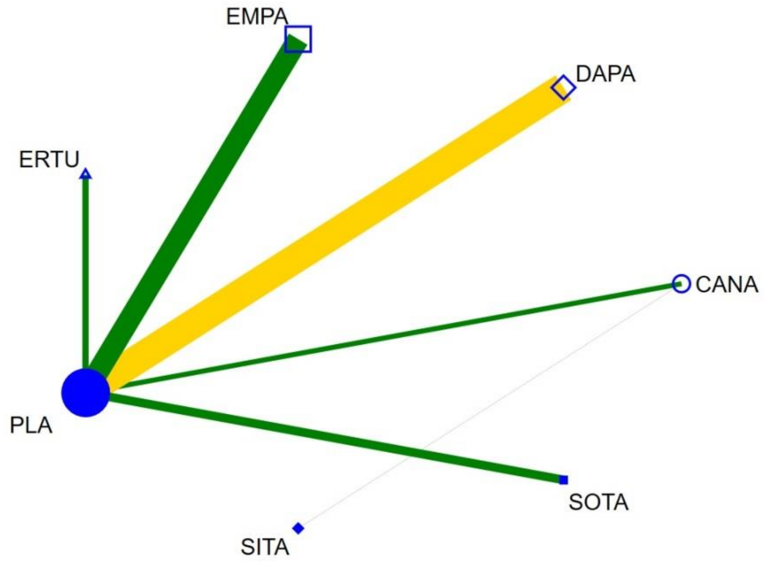

b) all-cause mortality

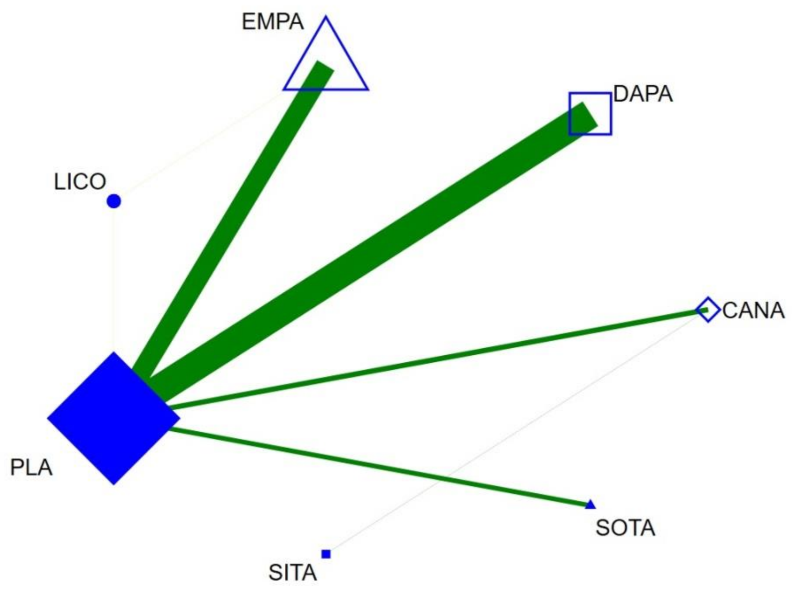

c) CV mortality

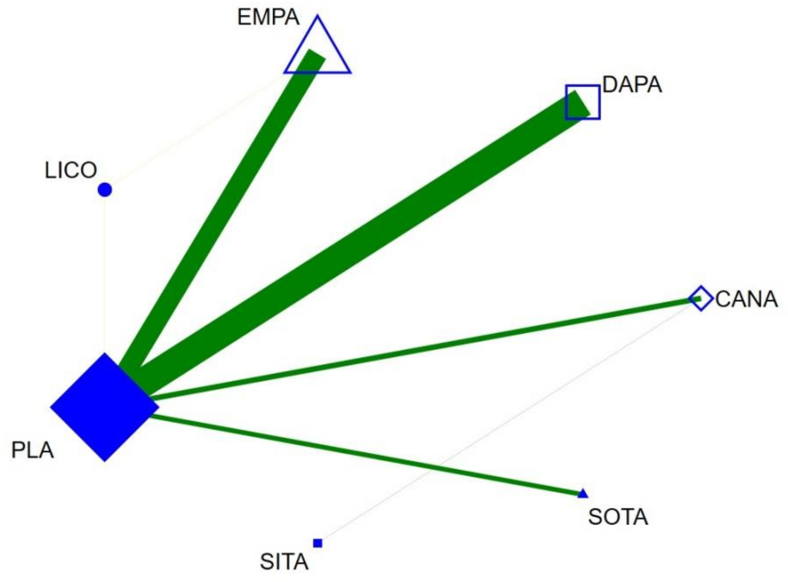

d) hospitalization for HF

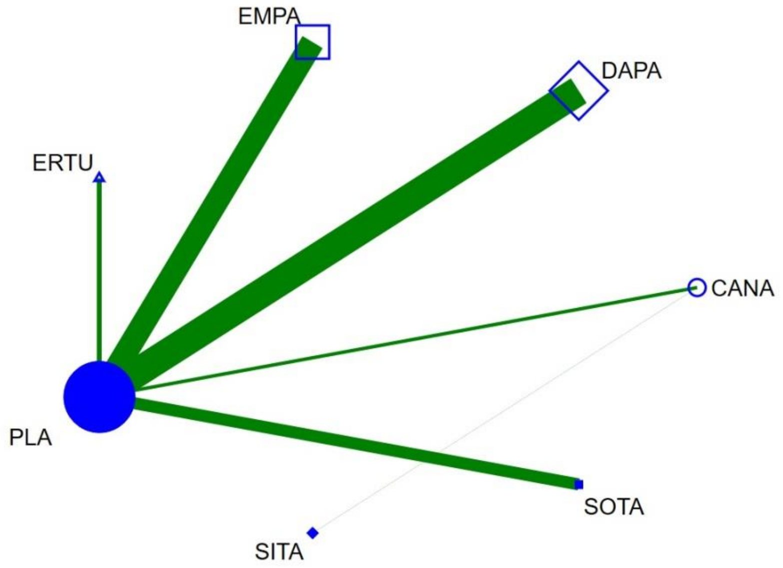

e) worsening RF

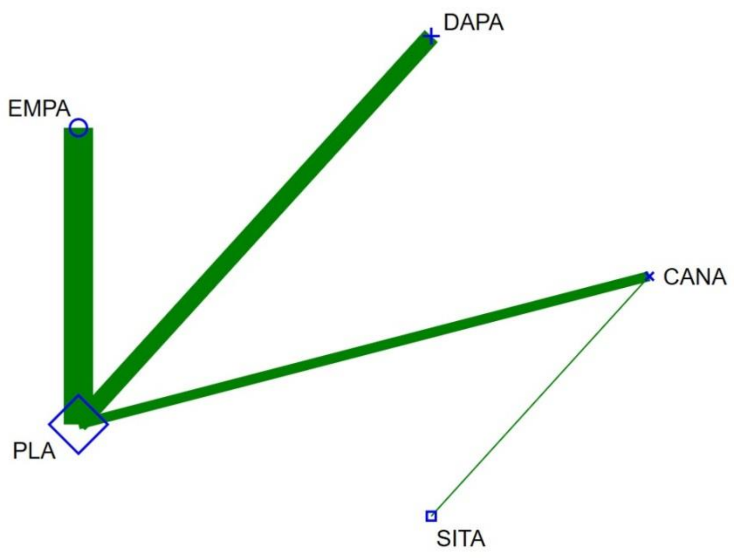

f) worsening RF or CV death

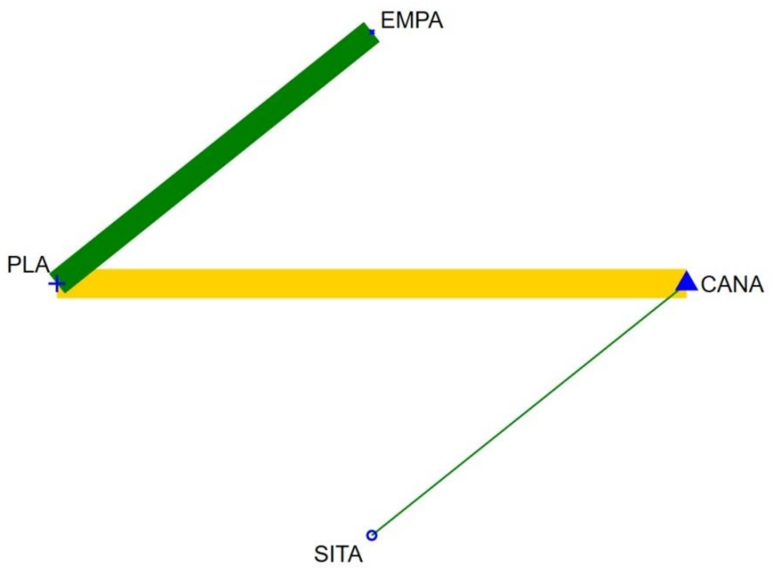

Legend: CANA, canagliflozin; CV, cardiovascular; DAPA, dapagliflozin; EMPA, empagliflozin; ERTU, ertugliflozin; HF, heart failure; LICO, licogliflozin; PLA, placebo; RF, renal function; SITA, sitagliptin; SOTA, sotagliflozin. Nodes and edges are weighted according to the number of trials including the respective interventions. Colored edges are employed to present

the risk of bias for each direct comparison in the network, with green, yellow and red colors being used to denote pairwise meta-analyses of low, unclear and high risk of bias.

Online Figure 3: Individual risk of bias assessment

Record	Trial [a]	Random sequence generation (selection bias)	Allocation concealment (selection bias)	Blinding of participants and personnel (performance bias)	Blinding of outcome assessment (detection bias)	Intention-to-treat analysis (attrition bias)	Incomplete outcome data (attrition bias)	Groups balanced at baseline (selection bias)	Selective reporting (reporting bias)	Independent funding	Trials stopped early	Prospective clinical trial registration
Bhatt (8)	SOLOIST-WHF	✓	✓	✓	✓	✓	✓	✓	✓	✗	✗	✓
Butler (25)<br>Fitchett (26,27)	EMPA-REG OUTCOME	✗	✓	✓	✓	✓	✓	✓	✓	✗	✓	✓
Cannon (28), Cosentino (29)	VERTIS CV	✗	✓	✓	✓	✓	!	✓	✓	✗	✓	✓
Carbone (30)	CANA-HF	✓	✓	✓	✓	✓	✓	✓	✓	✗	✓	✓
de Boer (31)		✓	✓	✓	✓	✓	!	✗	!	✗	✗	✓
Jensen (32)	EMPIRE-HF	✓	✓	✓	✓	✓	✓	✓	✓	✓	✓	✓
Kato (33)	DECLARE	✗	✓	✓	✓	✓	!	!	!	✗	✓	✓
Kosiborod (34)		✗	✓	✓	✓	✓	✓	✓	✓	✗	✓	✓
Lee (35)	SUGAR-DM-HF	✓	✓	✓	✓	✓	✓	✓	✓	✗	✓	✓
McMurray (6, 36), Petrie (37)	DAPA-HF	✓	✓	✓	✓	✓	✓	✓	✓	✗	✓	✓
Nassif (38)	DEFINE-HF	✓	✓	✓	✓	✓	✓	✓	✓	✗	✓	✓
Packer (7), Anker (39)	EMPEROR-Reduced	✓	✓	✓	✓	✓	✓	✓	✓	✗	✓	✓
Perkovic (40)	CREDENCE	✗	✓	✓	✓	✓	!	!	!	✗	✓	✓
Radholm (41)	CANVAS	✗	✓	✓	✓	✓	✓	✓	!	✗	✓	✓
Santos-Gallego (42)	EMPA-TROPISM	✓	✓	✓	✓	✓	✓	✓	✓	✓	✓	✓
Singh (43)	REFORM	✓	✓	✓	✓	✓	✓	✓	✓	✗	✓	✓
Boehringer Ingelheim (44)	EMPERIAL-Reduced	✓	✓	✓	✓	✓	✗	!	!	✗	✓	✓
Boehringer Ingelheim (45)	EMPERIAL-Preserved	✓	✓	✓	✓	✓	✗	!	!	✗	✓	✓

Legend: [a] Trial acronym/ short title.

Online Figure 4: Comparison funnel plot for the composite of hospitalization for HF or CV death

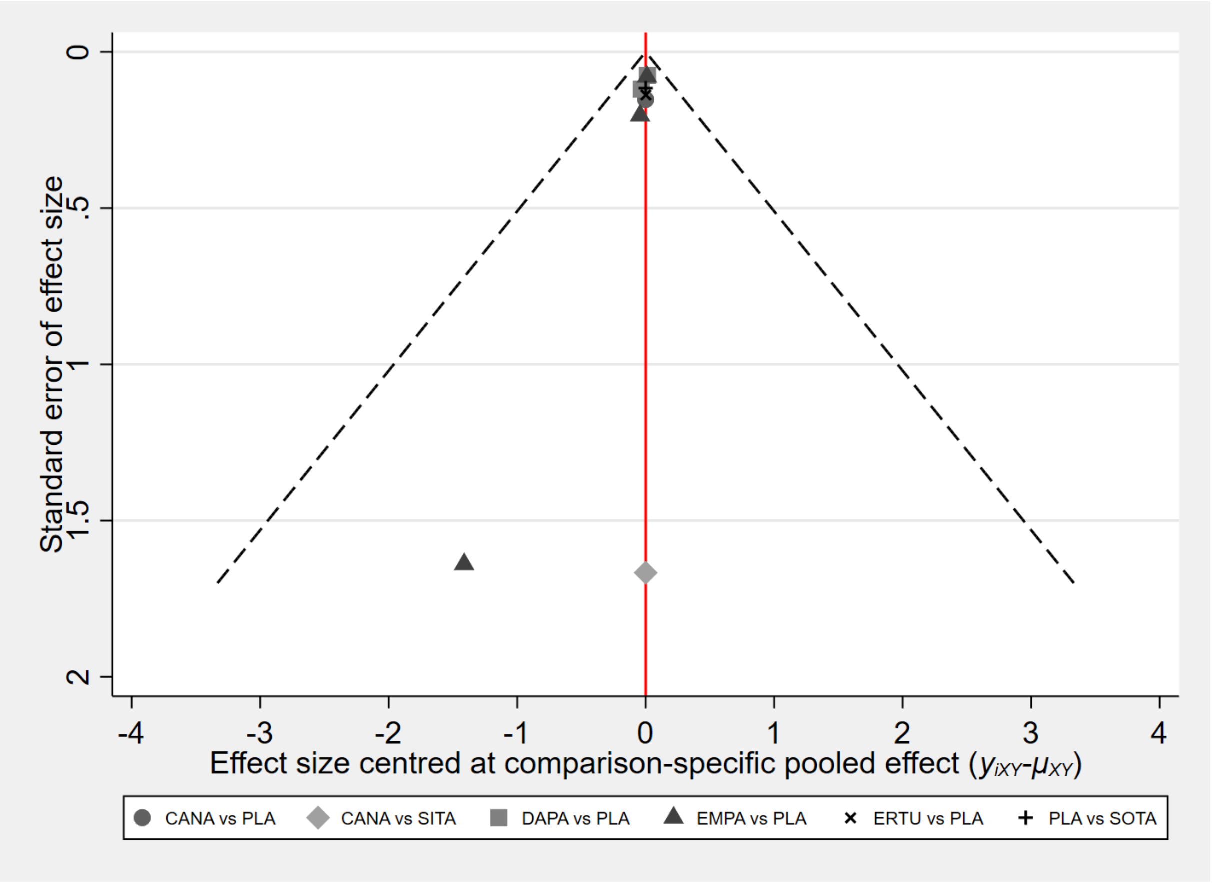

Legend: CANA, canagliflozin; CV, cardiovascular; DAPA, dapagliflozin; EMPA, empagliflozin; ERTU, ertugliflozin; HF, heart failure; PLA, placebo; SITA, sitagliptin; SOTA, sotagliflozin.

Online Figure 5: Predictive interval plot of individual SGLT2i for hospitalization all-cause mortality

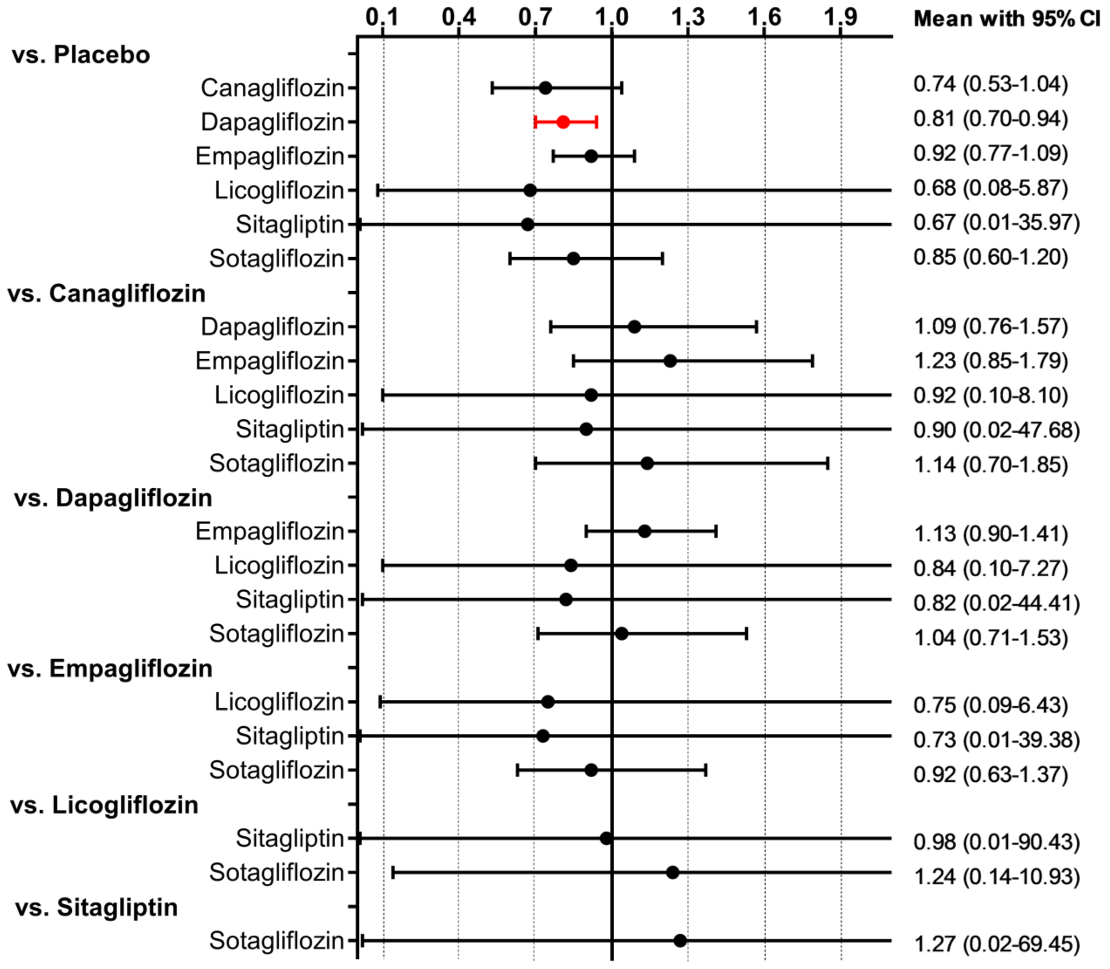

Legend: CI, confidence interval; SGLT2i, sodium-glucose cotransporter 2 inhibitor. The predictive interval plot represents a forest plot of the joint estimated summary effects from both direct and indirect comparisons along with their confidence intervals. Significant summary effects are shown in red.

Online Figure 6: Graphical ranking of SGLT2i based on SUCRA values (all-cause mortality)

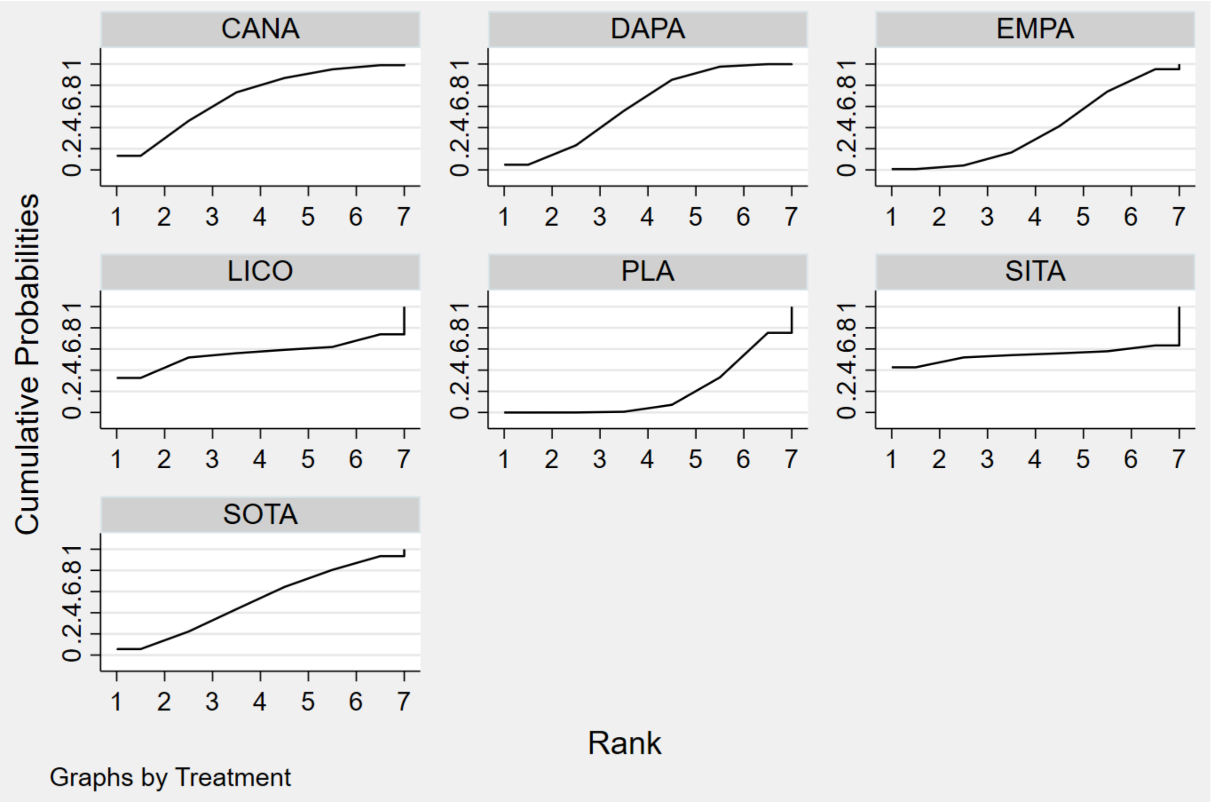

Legend: CANA, canagliflozin; DAPA, dapagliflozin; EMPA, empagliflozin, LICO, licogliflozin; PLA, placebo, SITA, sitagliptin, SOTA, sotagliflozin.

Online Figure 7: Predictive interval plot of SGLT2i classes for all-cause mortality

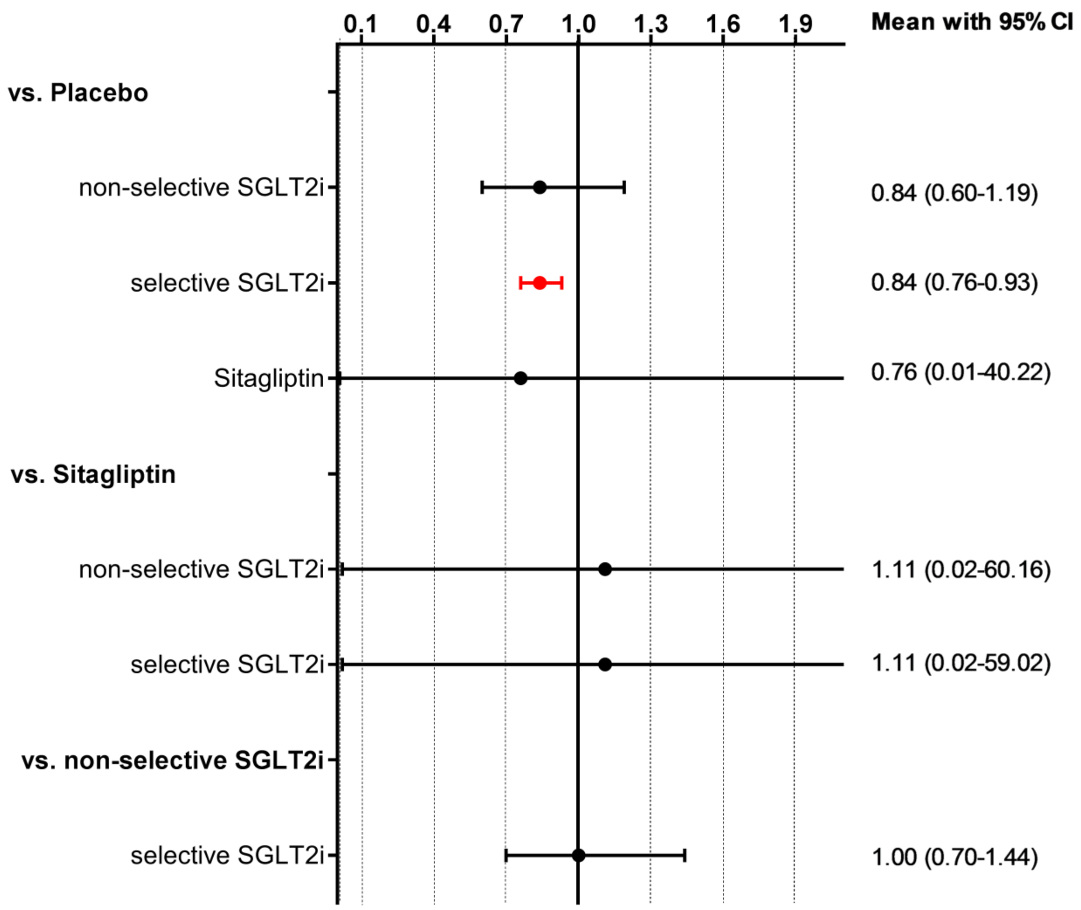

Legend: CI, confidence interval; SGLT2i, sodium-glucose cotransporter 2 inhibitor. The predictive interval plot represents a forest plot of the joint estimated summary effects from both direct and indirect comparisons along with their confidence intervals. Significant summary effects are shown in red.

Online Figure 8: Relationship between effect size and receptor selectivity of SGLT2i for all-cause mortality

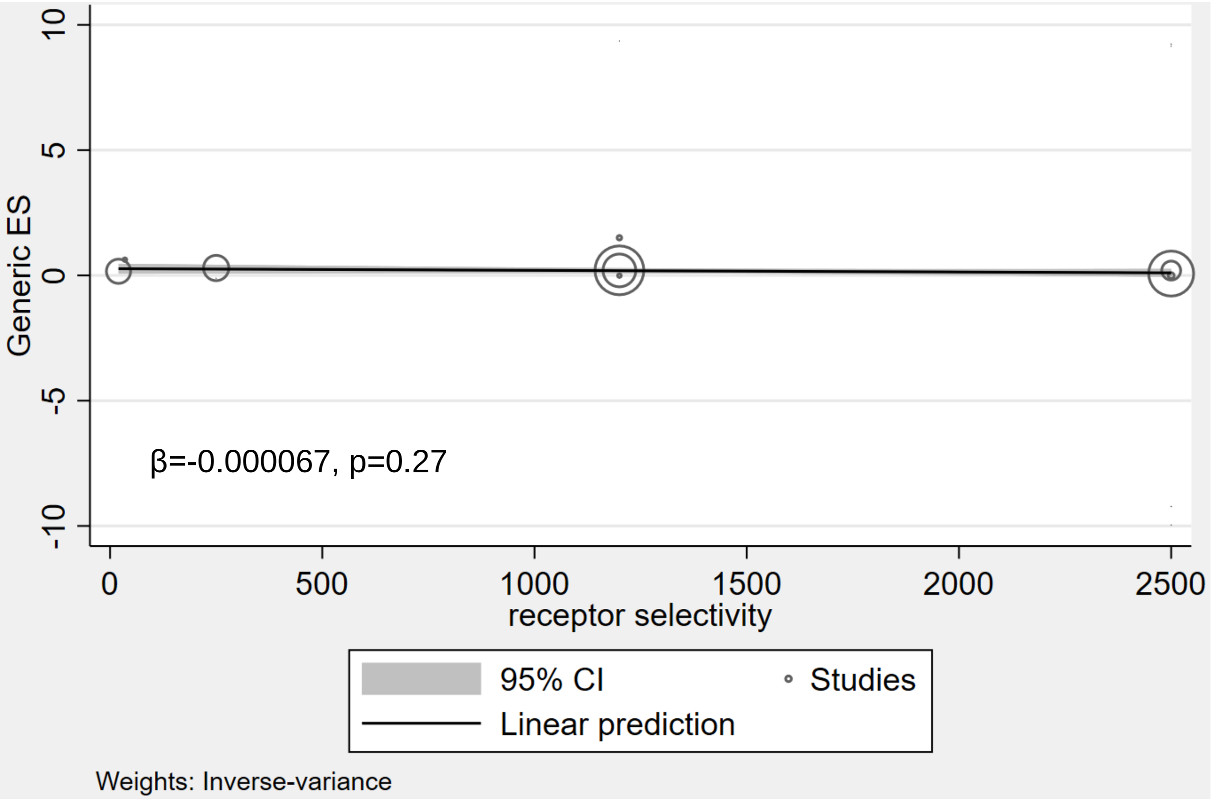

Legend: CI, confidence interval; ES, effect size; SGLT2i, sodium-glucose cotransporter 2 inhibitor. Each bubble represents a SGLT2i trial. The symbol size represents the sample size of the respective trials.

Online Figure 9: Predictive interval plot of individual SGLT2i classes for CV mortality

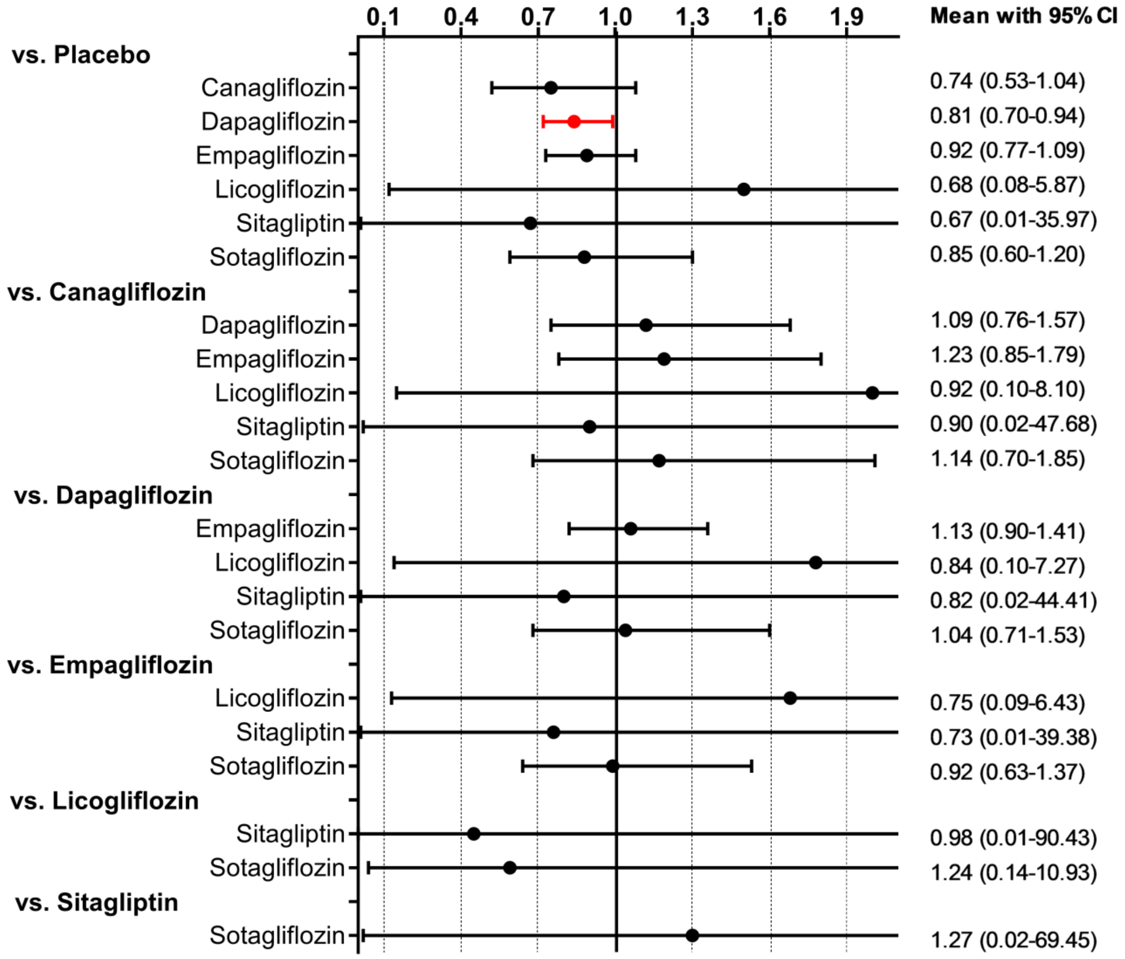

Legend: CI, confidence interval; CV, cardiovascular, SGLT2i, sodium-glucose cotransporter 2 inhibitor

Online Figure 10: Graphical ranking of SGLT2i based on SUCRA values (CV mortality)

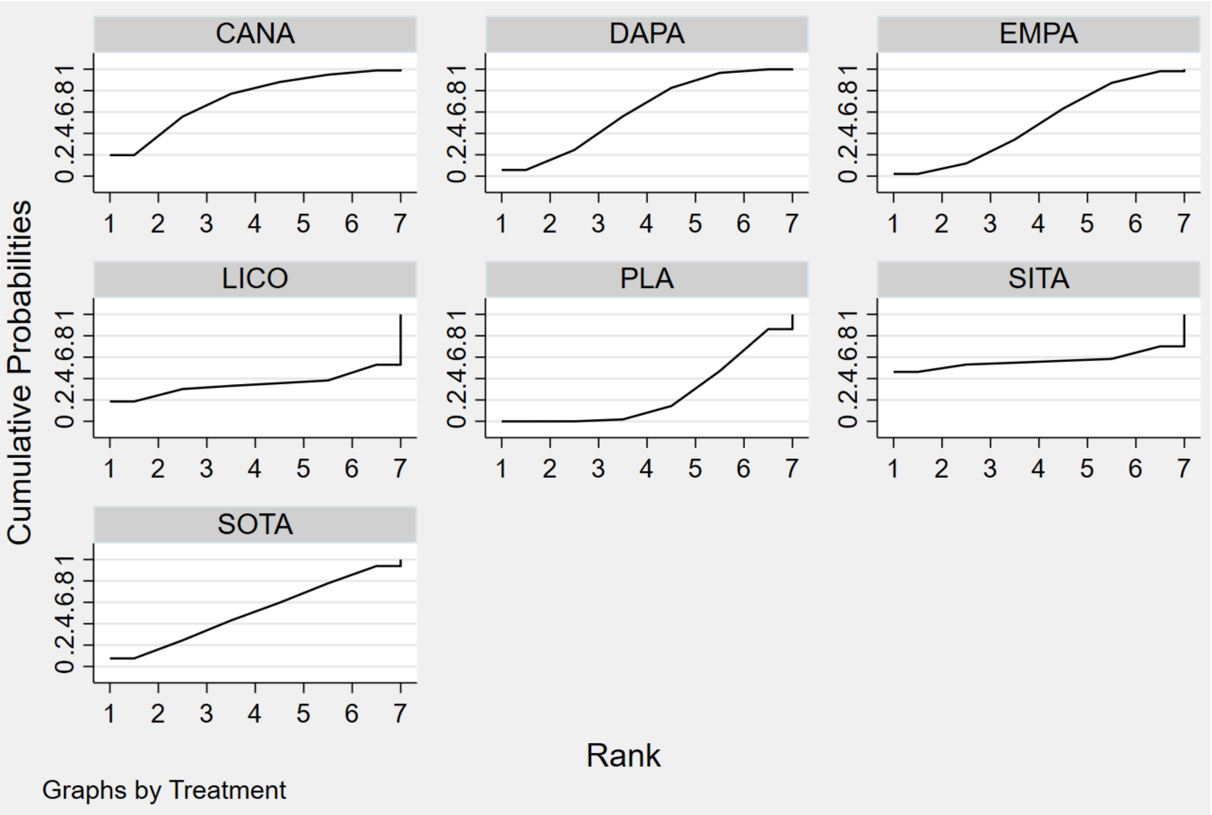

Legend: CANA, canagliflozin; CV, cardiovascular; DAPA, dapagliflozin; EMPA, empagliflozin, LICO, licogliflozin; PLA, placebo; SITA, sitagliptin, SOTA, sotagliflozin.

Online Figure 11: Predictive interval plot of SGLT2i classes for CV mortality

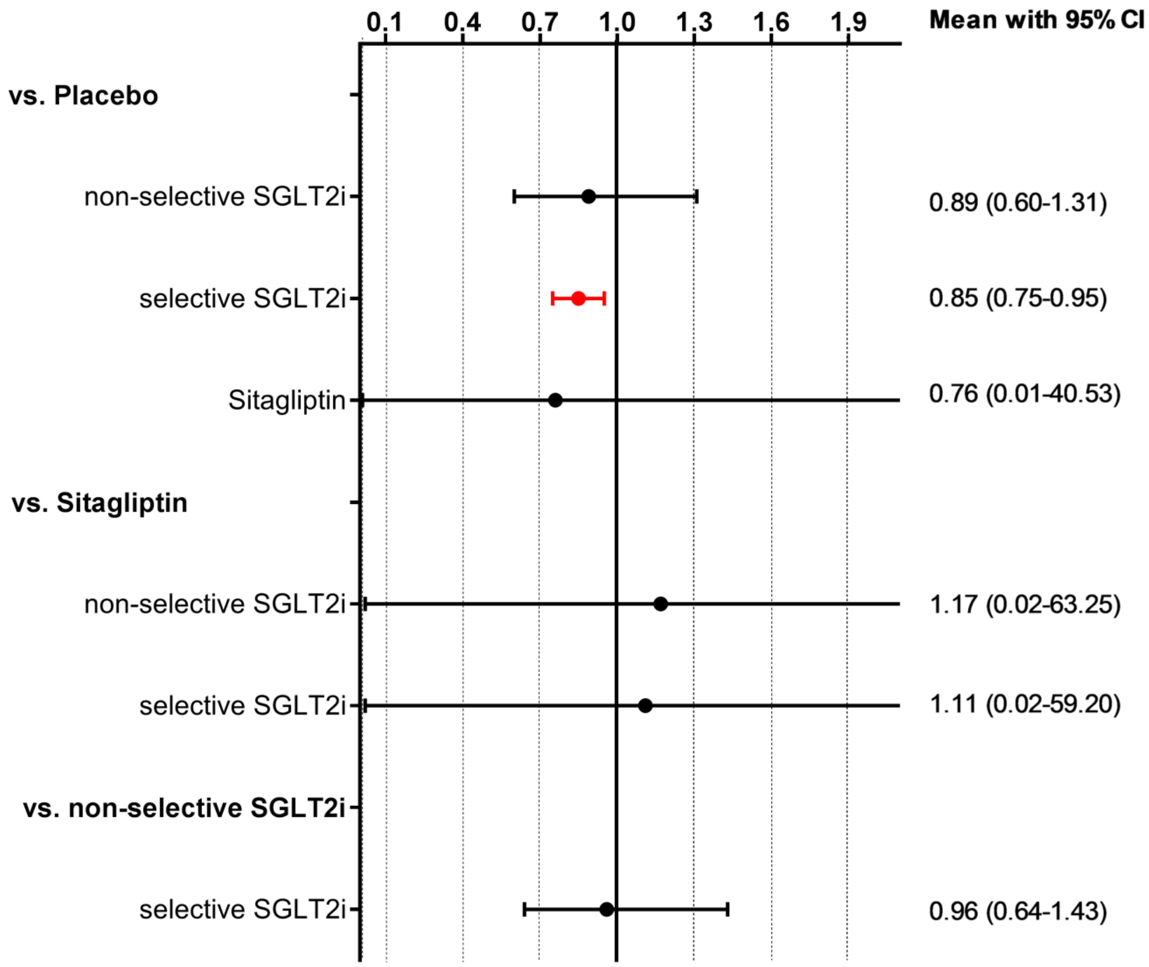

Legend: CI, confidence interval; CV, cardiovascular; SGLT2i, sodium-glucose cotransporter 2 inhibitor

Online Figure 12: Relationship between effect size and receptor selectivity of SGLT2i for CV mortality

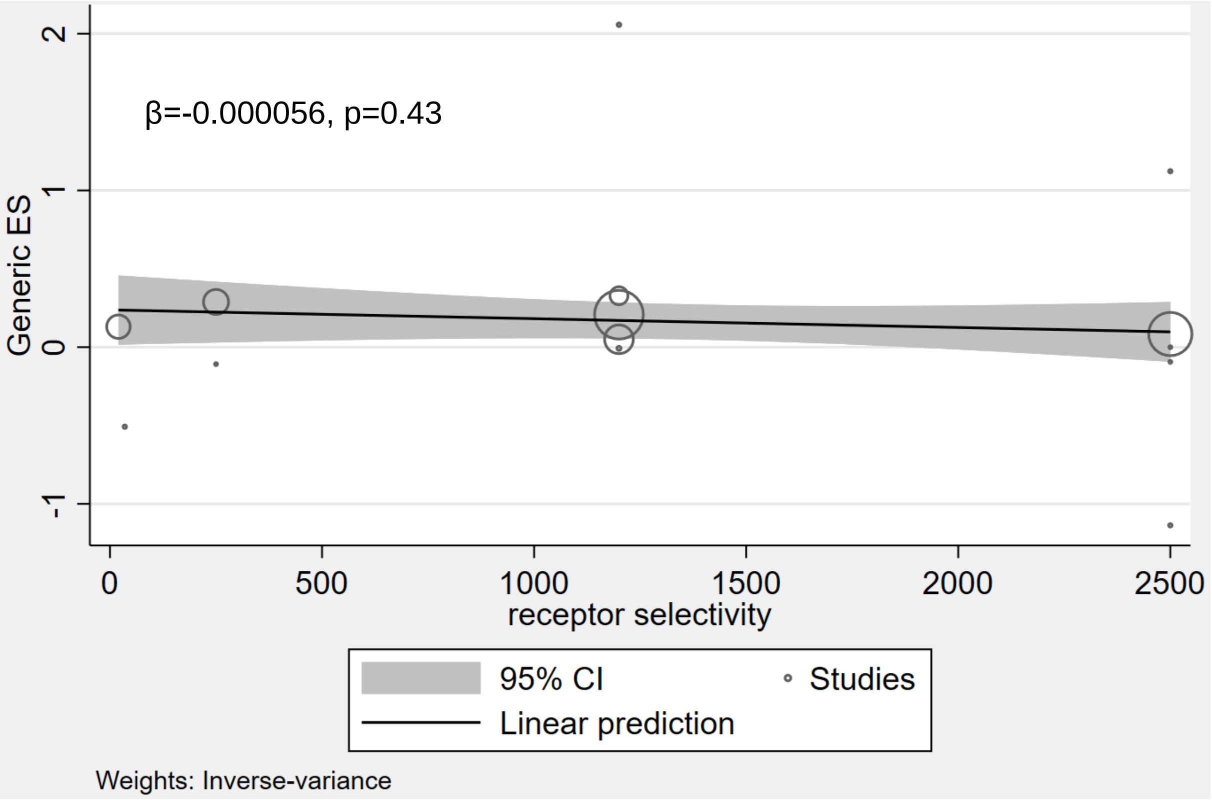

Legend: CI, confidence interval; CV, cardiovascular; ES, effect size.

Online Figure 13: Predictive interval plot of individual SGLT2i for hospitalization for HF

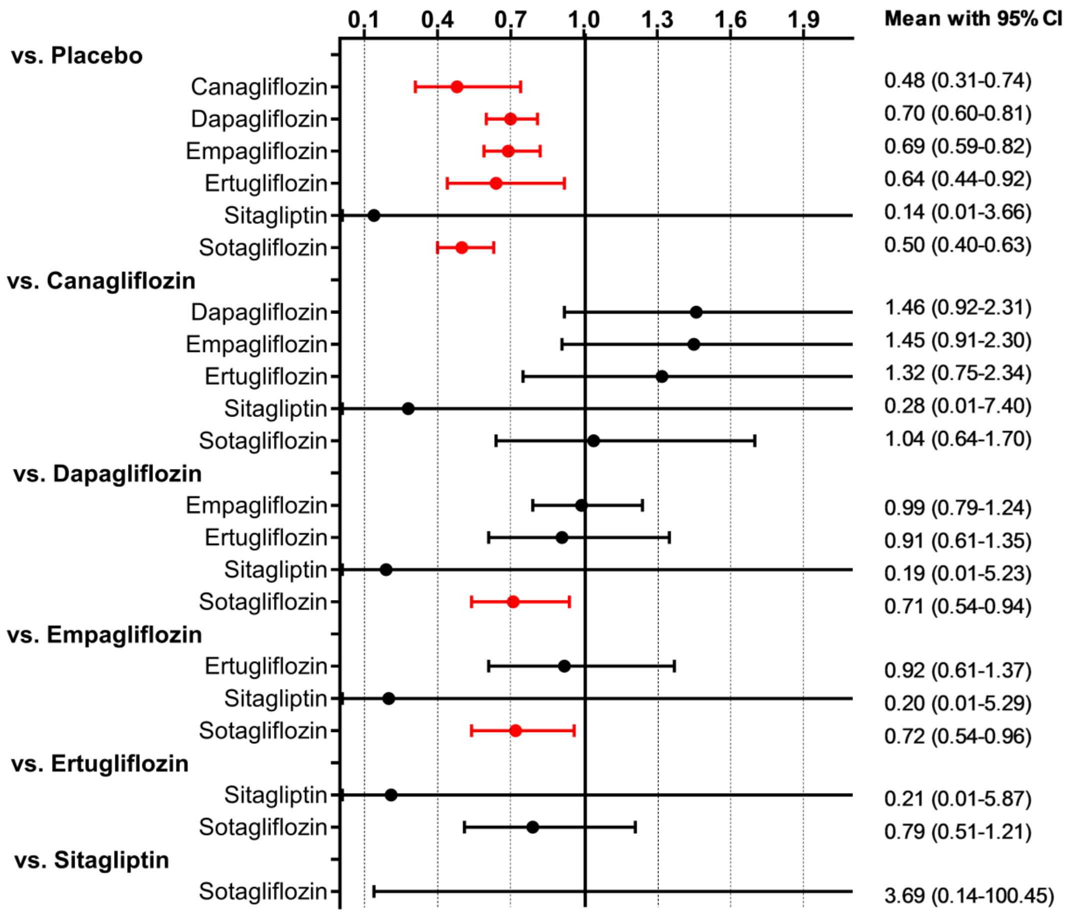

Legend: CI, confidence interval; HF, heart failure; SGLT2i, sodium-glucose cotransporter 2 inhibitor.

Online Figure 14: Graphical ranking of SGLT2i based on SUCRA values (hospitalization for HF)

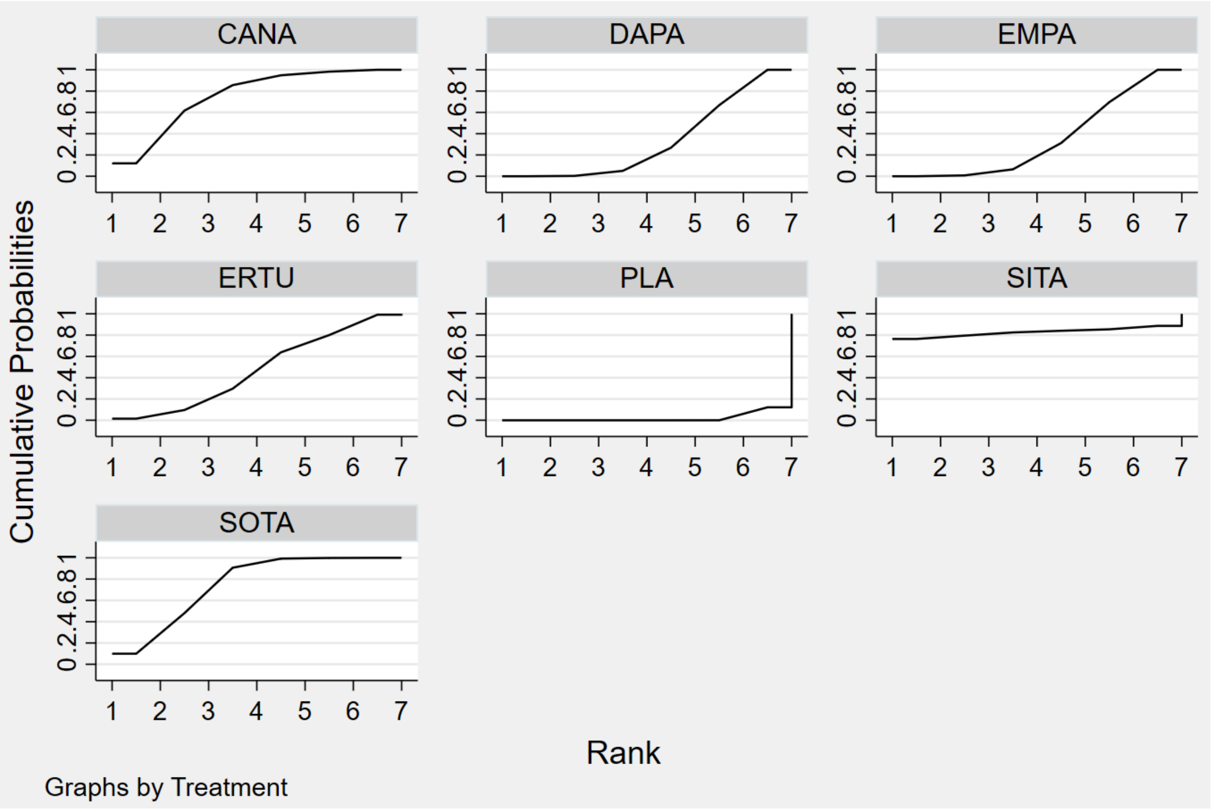

Legend: CANA, canagliflozin; DAPA, dapagliflozin; EMPA, empagliflozin, ERTU, ertugliflozin; HF, heart failure, PLA, placebo; SITA, sitagliptin, SOTA, sotagliflozin.

Online Figure 15: Predictive interval plot of SGLT2i classes for hospitalizations for HF

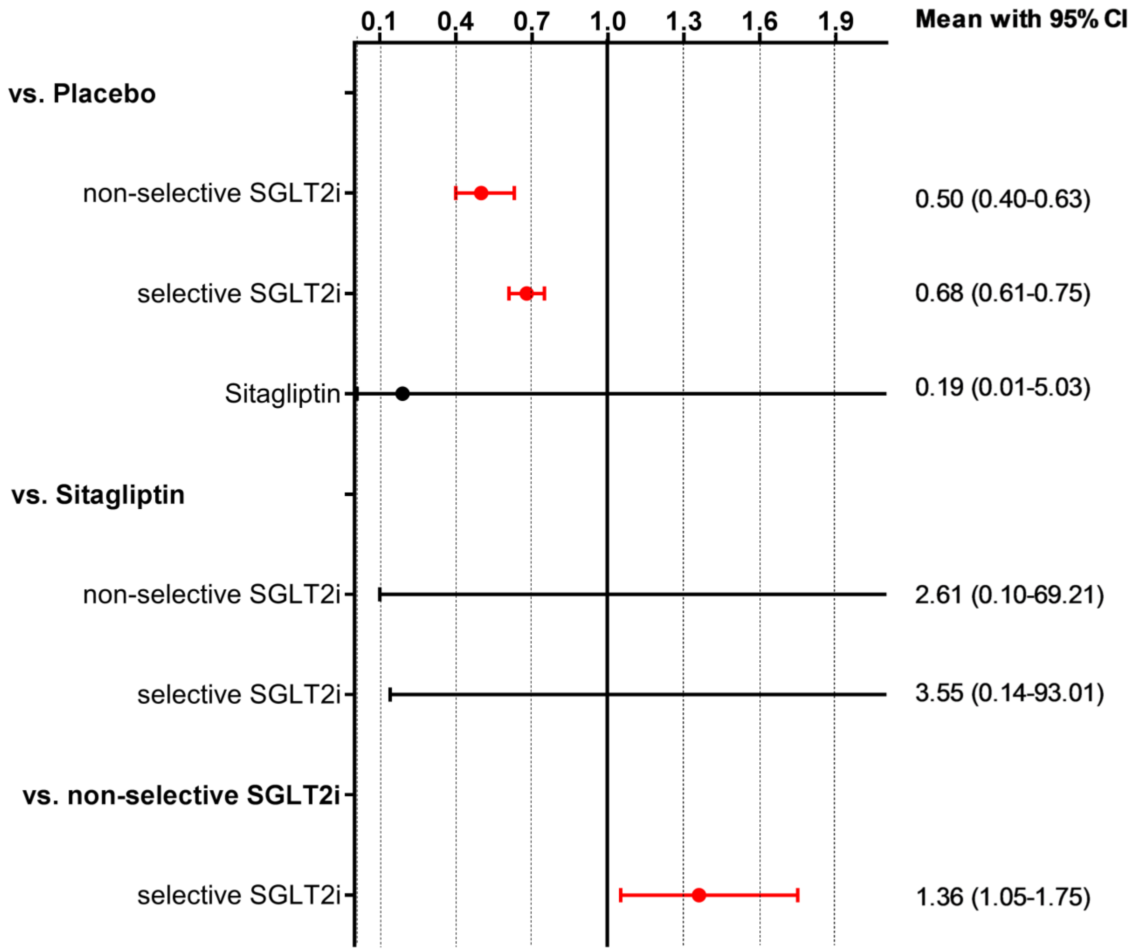

Legend: CI, confidence interval; HF, heart failure; SGLT2i, sodium-glucose cotransporter 2 inhibitor.

Online Figure 16: Relationship between effect size and receptor selectivity of SGLT2i for hospitalizations for HF

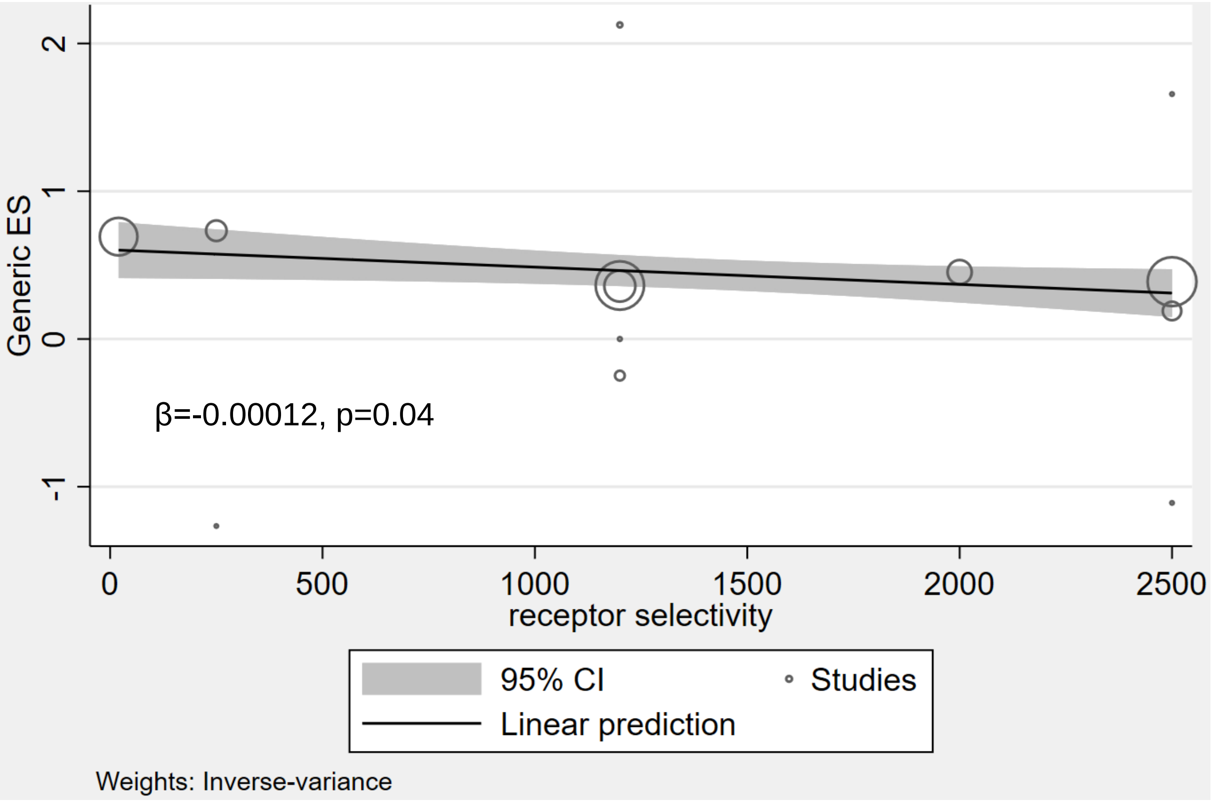

Legend: CI, confidence interval; ES, effect size; SGLT2i, sodium-glucose cotransporter 2 inhibitor.

Online Figure 17: Predictive interval plot for worsening RF

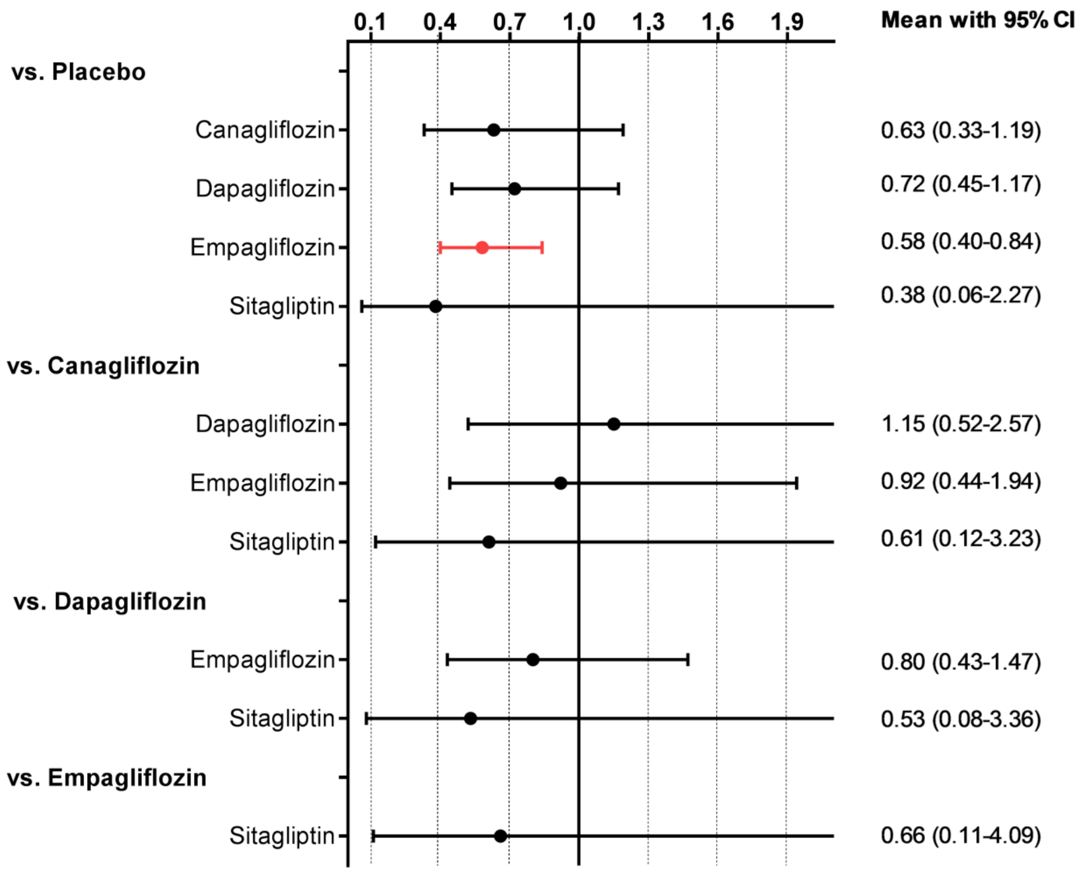

Legend: CI, confidence interval; RF, renal function.

Online Figure 18: Graphical ranking of SGLT2i based on SUCRA values (worsening RF)

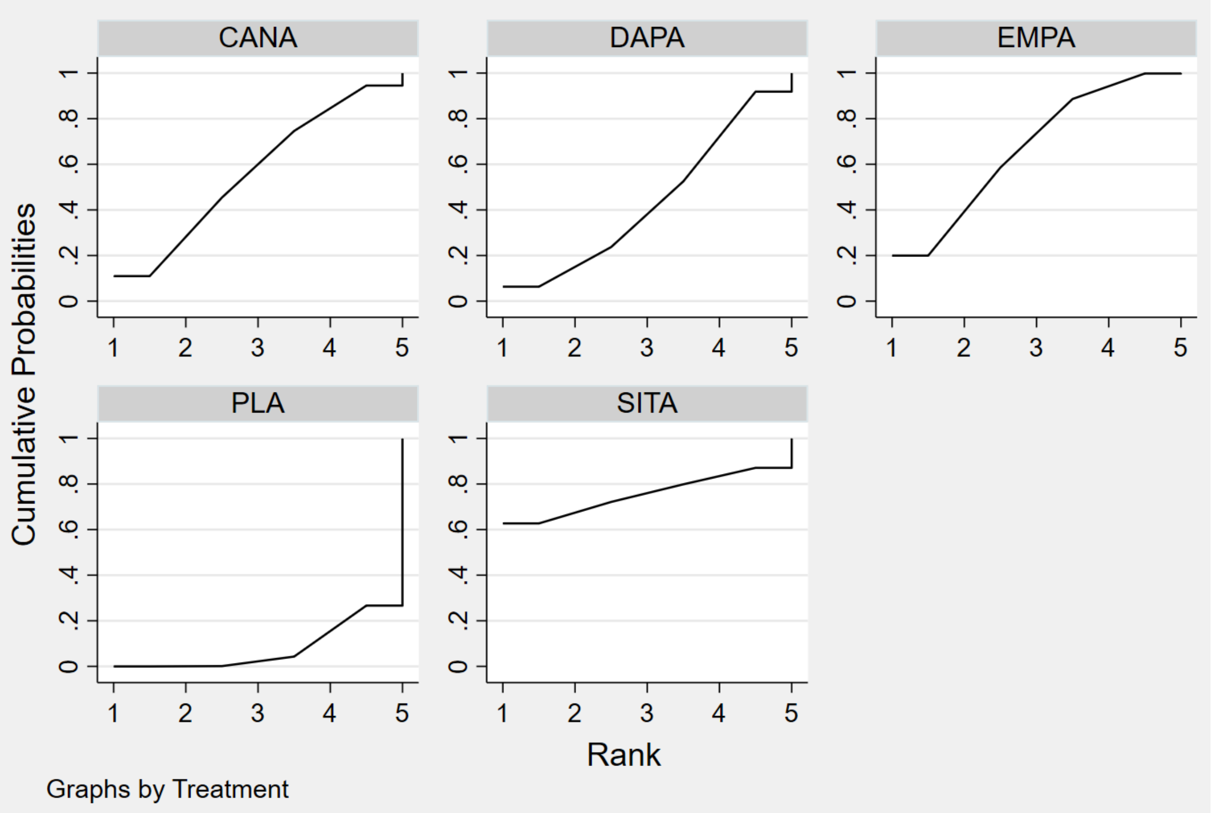

Legend: CANA, canagliflozin; DAPA, dapagliflozin; EMPA, empagliflozin, RF, renal function, PLA, placebo; SITA, sitagliptin

Online Figure 19: Relationship between effect size and receptor selectivity of SGLT2i for worsening RF

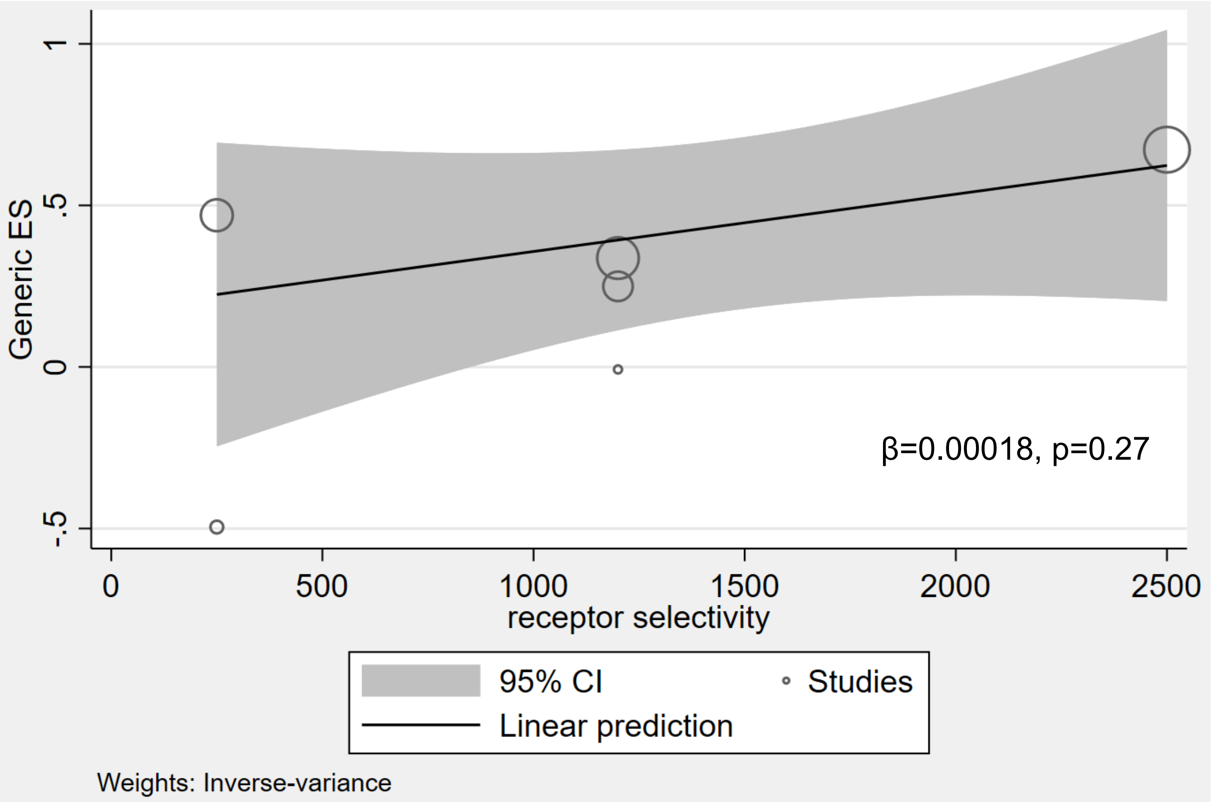

Legend: CI, confidence interval; EF, effect size; RF, renal function.

Online Figure 20: Predictive interval plot for worsening RF or CV death

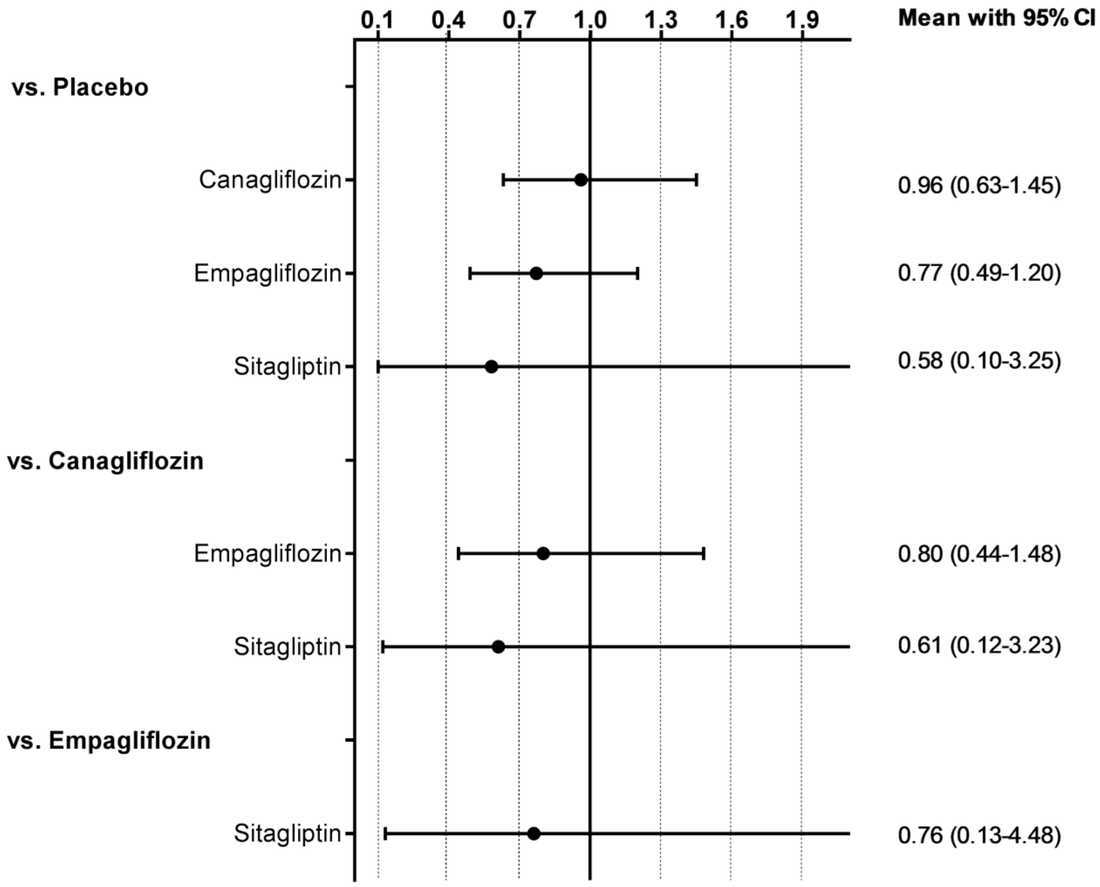

Legend: CI, confidence interval; CV, cardiovascular; RF, renal function.

Online Figure 21: Predictive interval plot for hospitalization for HF or CV death in subgroups of patients with both HF and T2D (sensitivity analysis)

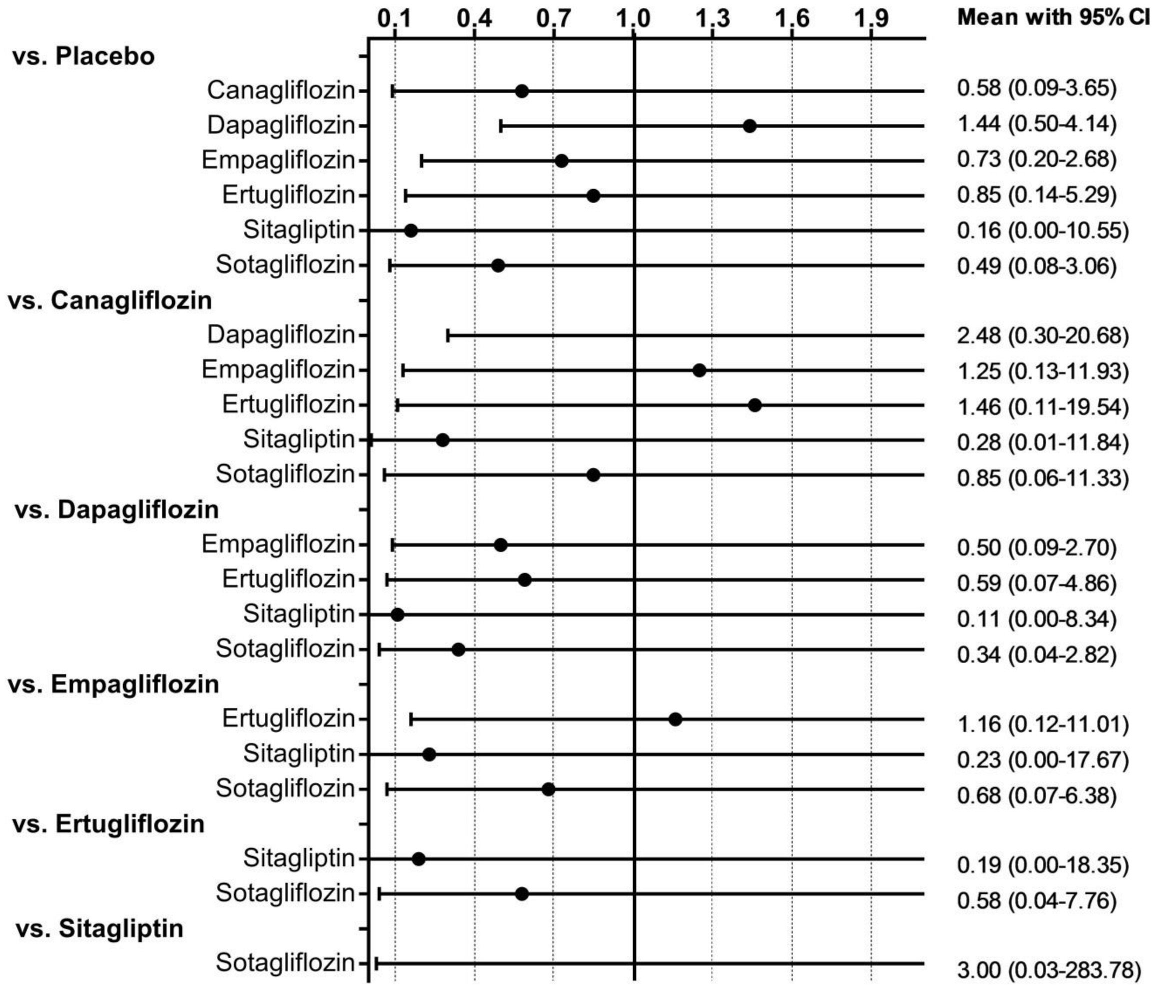

Legend: CV, cardiovascular; HF, heart failure; T2D, type 2 diabetes mellitus

Online Figure 22: Predictive interval plot for SGLT2i classes with respect to hospitalization for HF or CV death in subgroups of patients with both HF and T2D (sensitivity analysis)

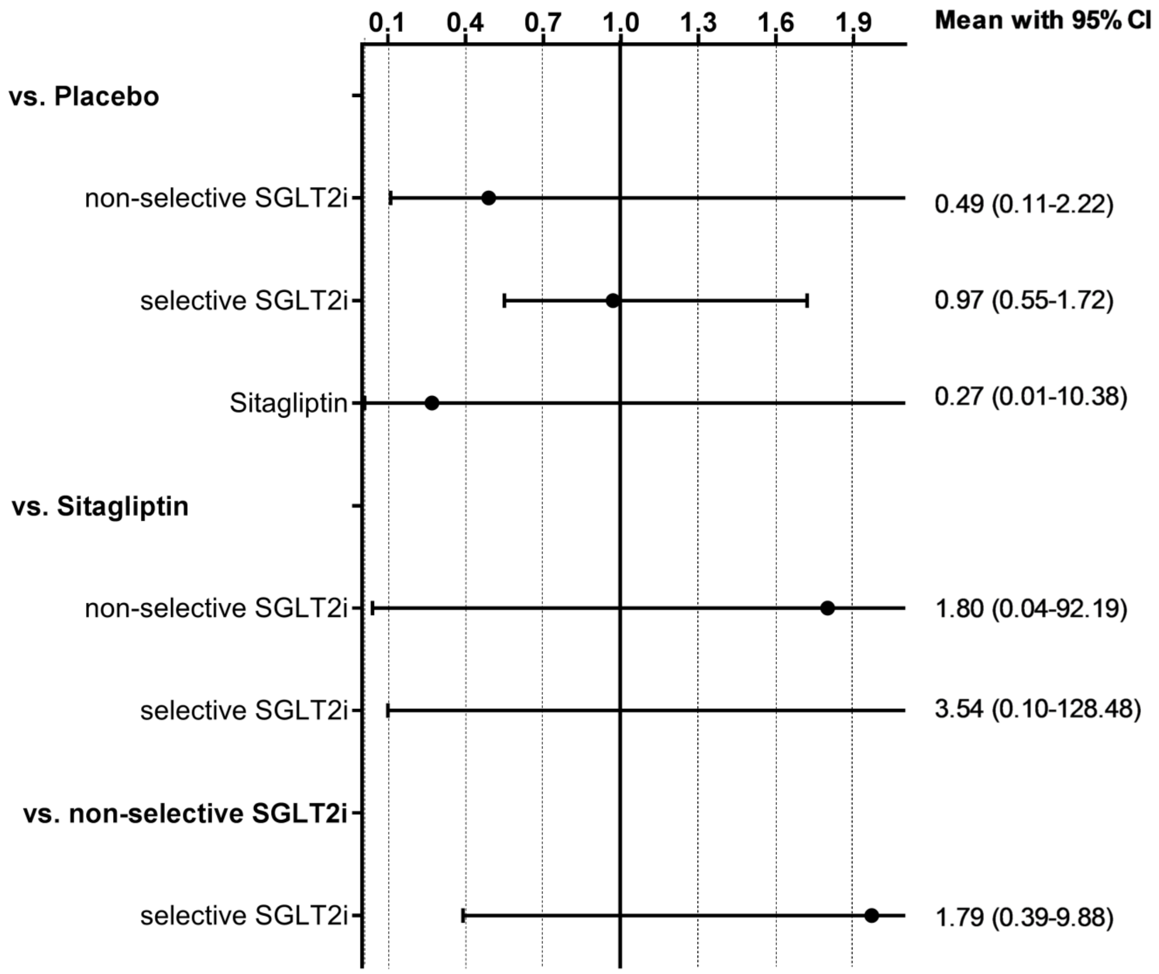

Legend: CI, confidence interval; CV, cardiovascular; HF, heart failure, T2D, type 2 diabetes mellitus; SGLT2i, sodium-glucose cotransporter 2 inhibitor.

Online Figure 23: Predictive interval plot for all-cause mortality in subgroups of patients with both HF and T2D (sensitivity analysis)

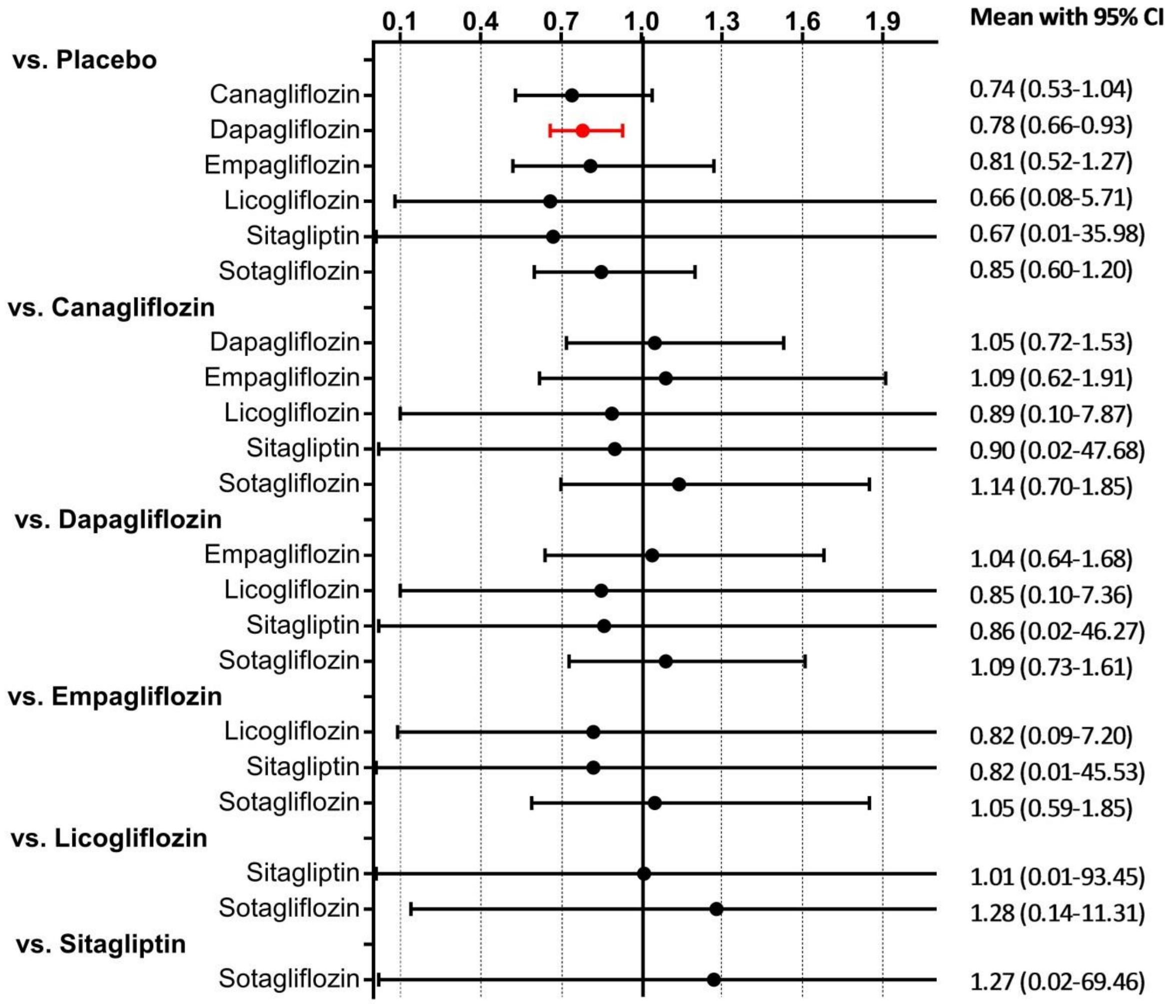

Legend: CI, confidence interval; HF, heart failure; T2D, type 2 diabetes mellitus. Significant summary effects are shown in red.

Online Figure 24: Predictive interval plot for SGLT2i classes with respect to all-cause mortality in subgroups of patients with both HF and T2D (sensitivity analysis)

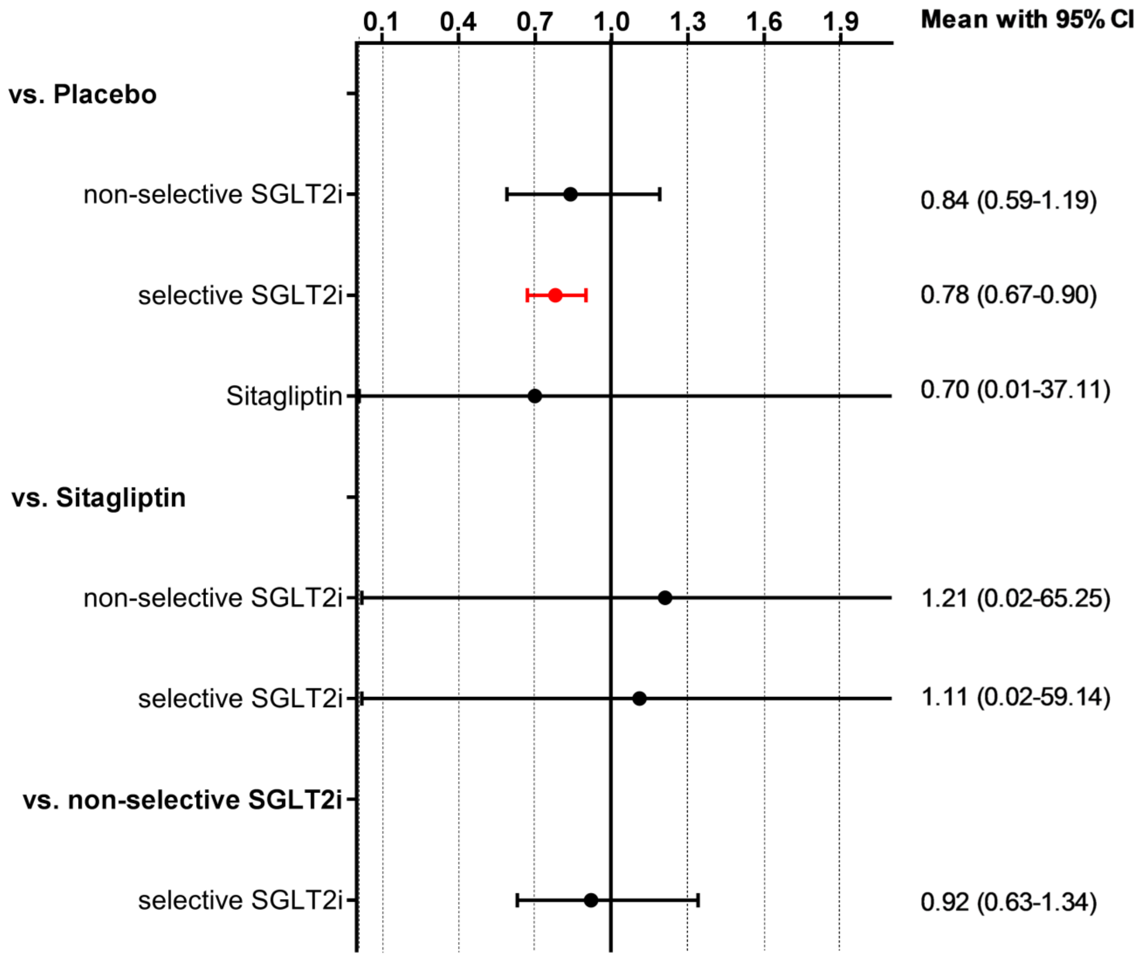

Legend: CI, confidence interval; HF, heart failure; SGLT2i, sodium-glucose cotransporter 2 inhibitor; T2D, type 2 diabetes mellitus. Significant results are shown in red color.

Online Figure 25: Predictive interval plot for CV mortality in subgroups of patients with both HF and T2D (sensitivity analysis)

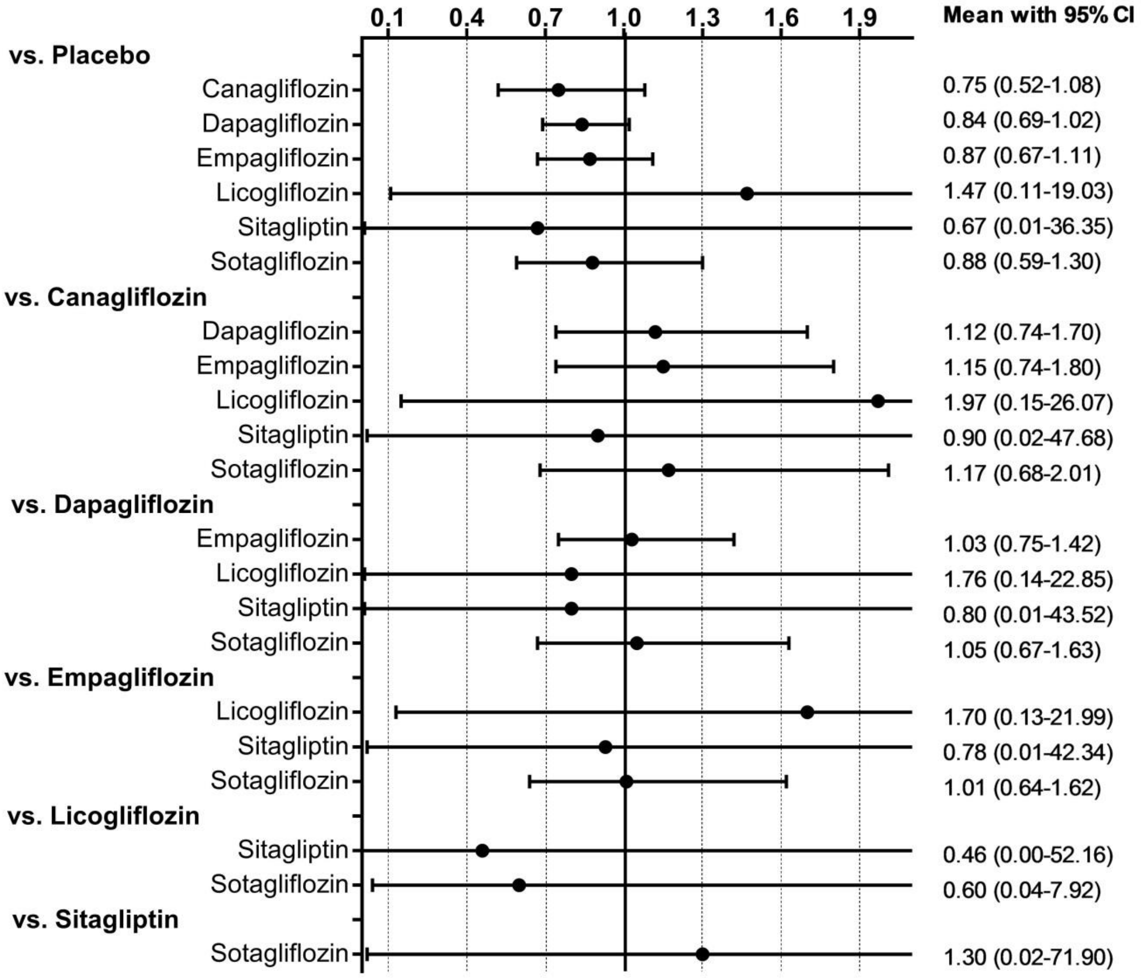

Legend: CI, confidence interval; HF, heart failure; T2D, type 2 diabetes mellitus

Online Figure 26: Predictive interval plot for SGLT2i classes with respect to CV mortality in subgroups of patients with both HF and T2D (sensitivity analysis)

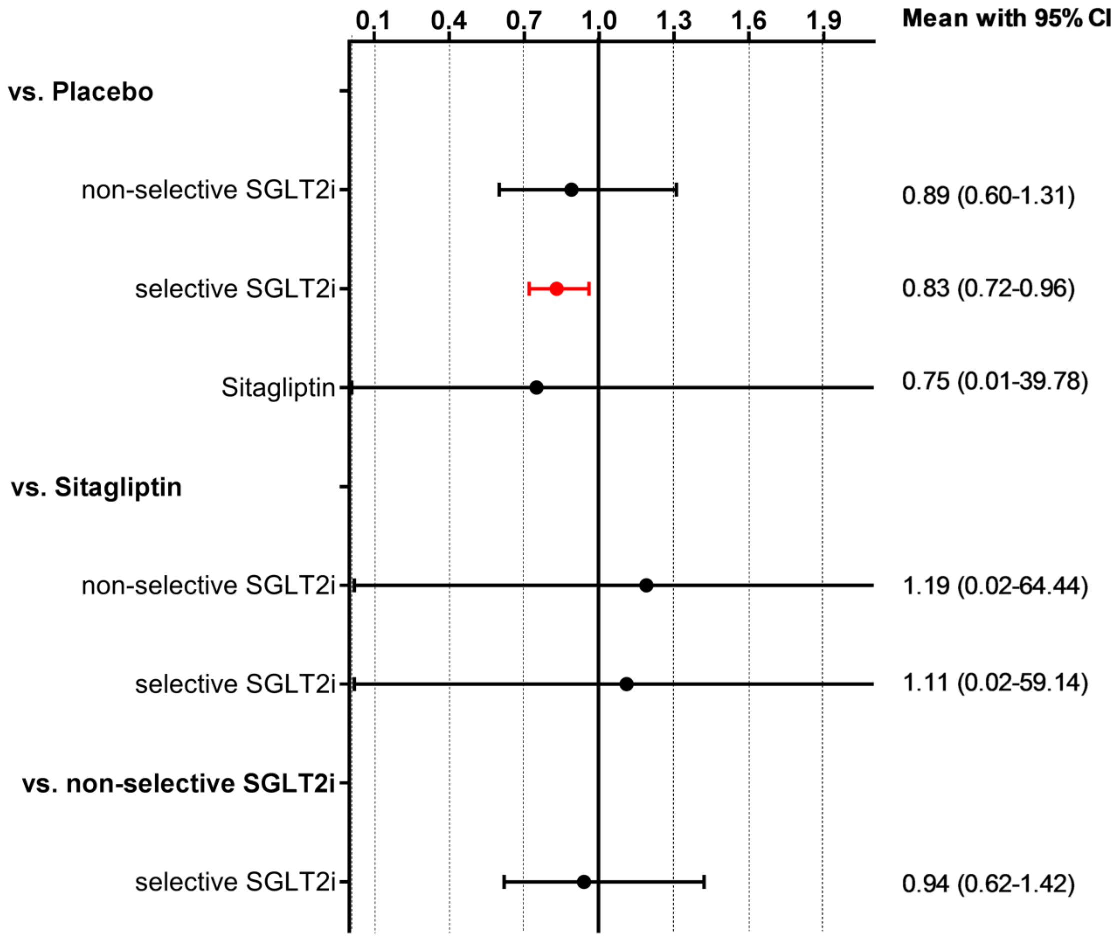

Legend: CI, confidence interval; CV, cardiovascular; HF, heart failure, SGLT2i, sodium-glucose cotransporter 2 inhibitor; T2D, type 2 diabetes mellitus. Significant results are shown in red color.

Online Figure 27: Predictive interval plot for hospitalization for HF in subgroups of patients with both HF and T2D (sensitivity analysis)

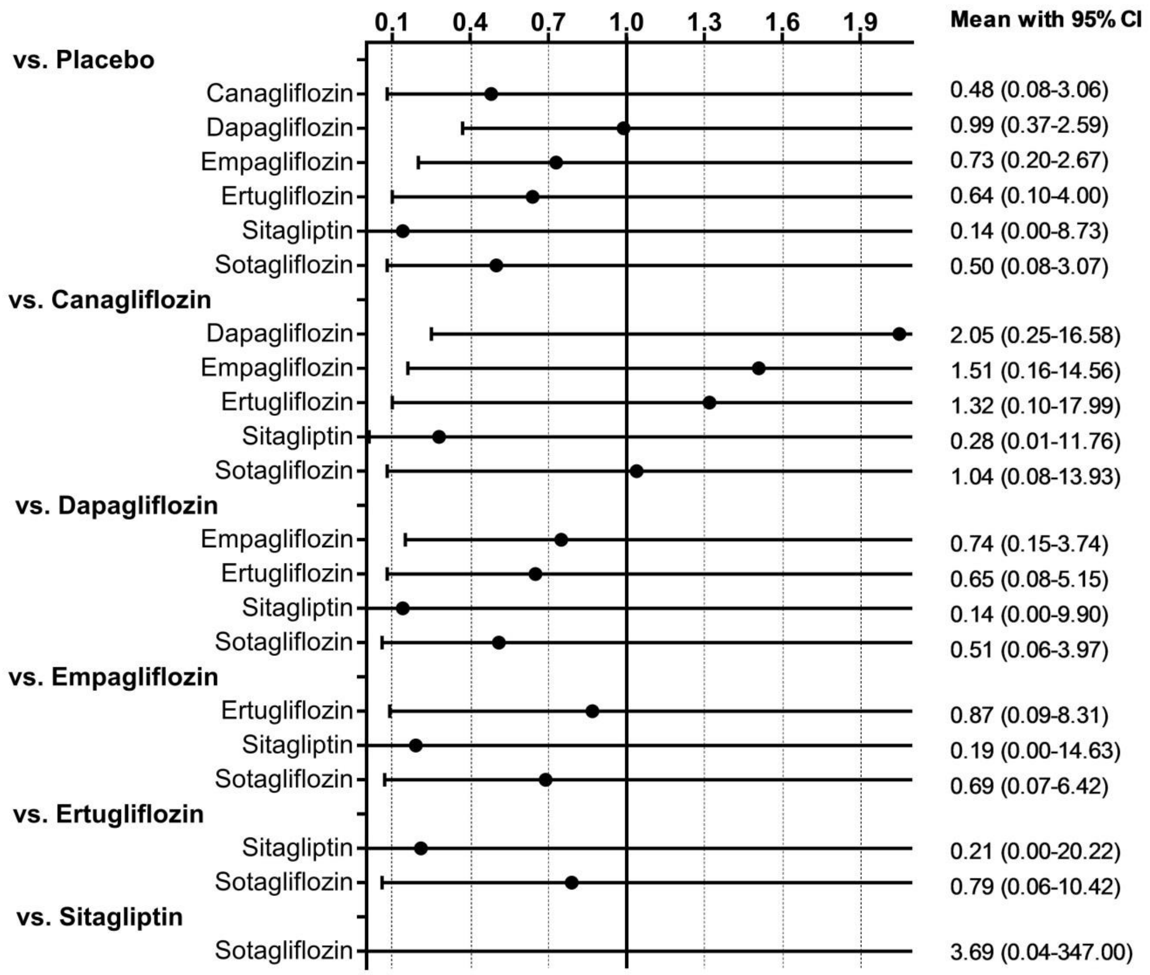

Legend: CI, confidence interval; HF, heart failure; T2D, type 2 diabetes mellitus

Online Figure 28: Predictive interval plot for SGLT2i classes with respect to hospitalizations for HF in subgroups of patients with both HF and T2D (sensitivity analysis)

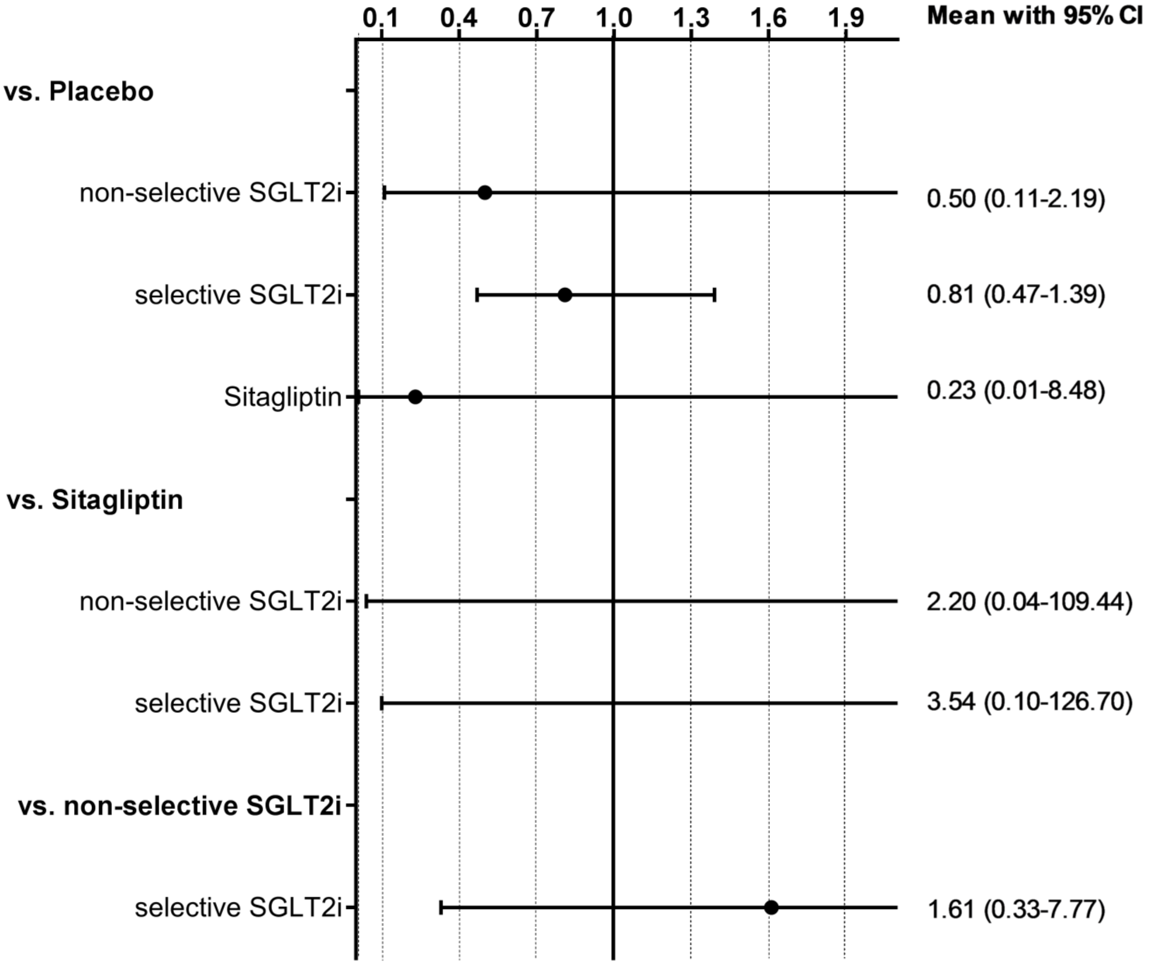

Legend: CI, confidence interval; HF, heart failure; SGLT2i, sodium-glucose cotransporter 2 inhibitor; T2D, type 2 diabetes mellitus.

Online Figure 29: Predictive interval plot for worsening RF in subgroups of patients with both HF and T2D (sensitivity analysis)

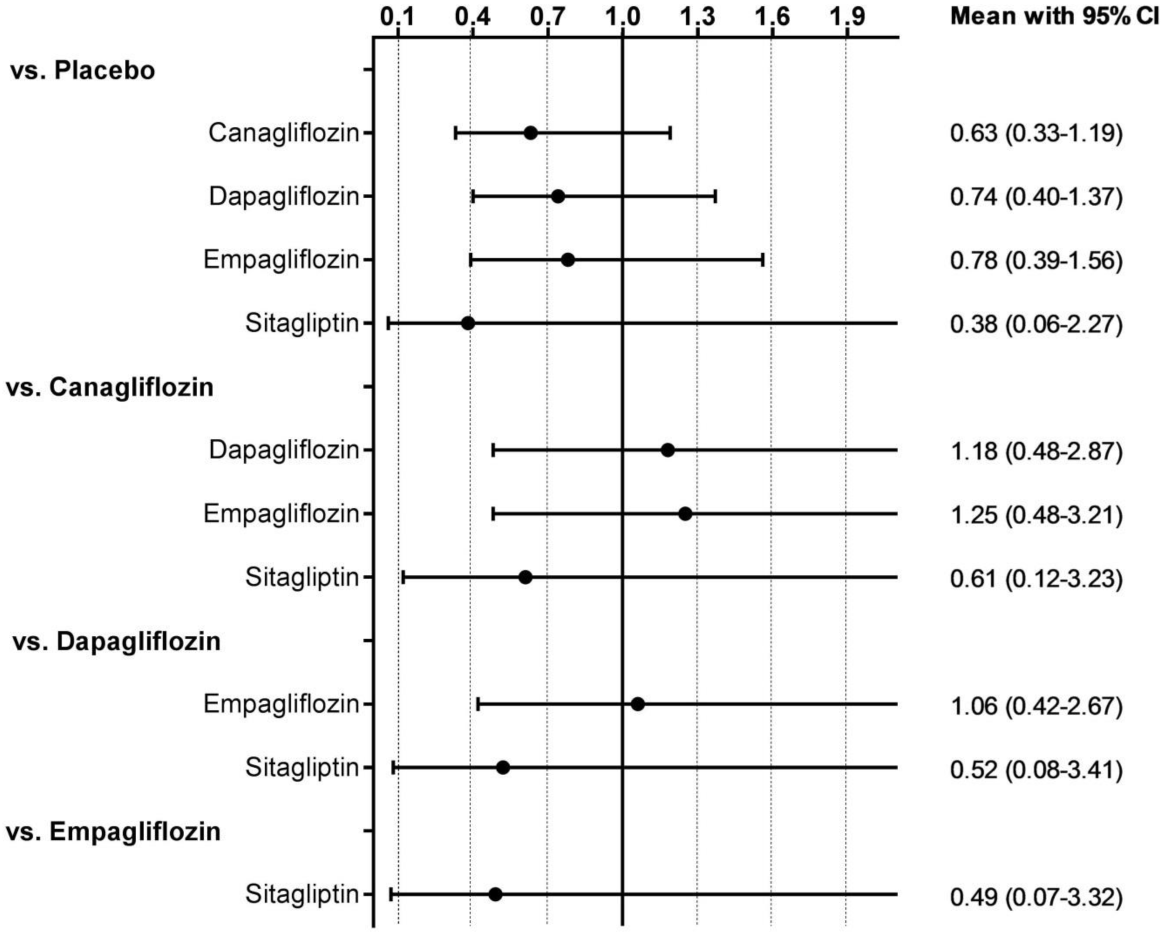

Legend: CI, confidence interval; HF, heart failure; RF, renal function; T2D, type 2 diabetes mellitus

Online Figure 30: Predictive interval plot for worsening RF or CV death in subgroups of patients with both HF and T2D (sensitivity analysis)

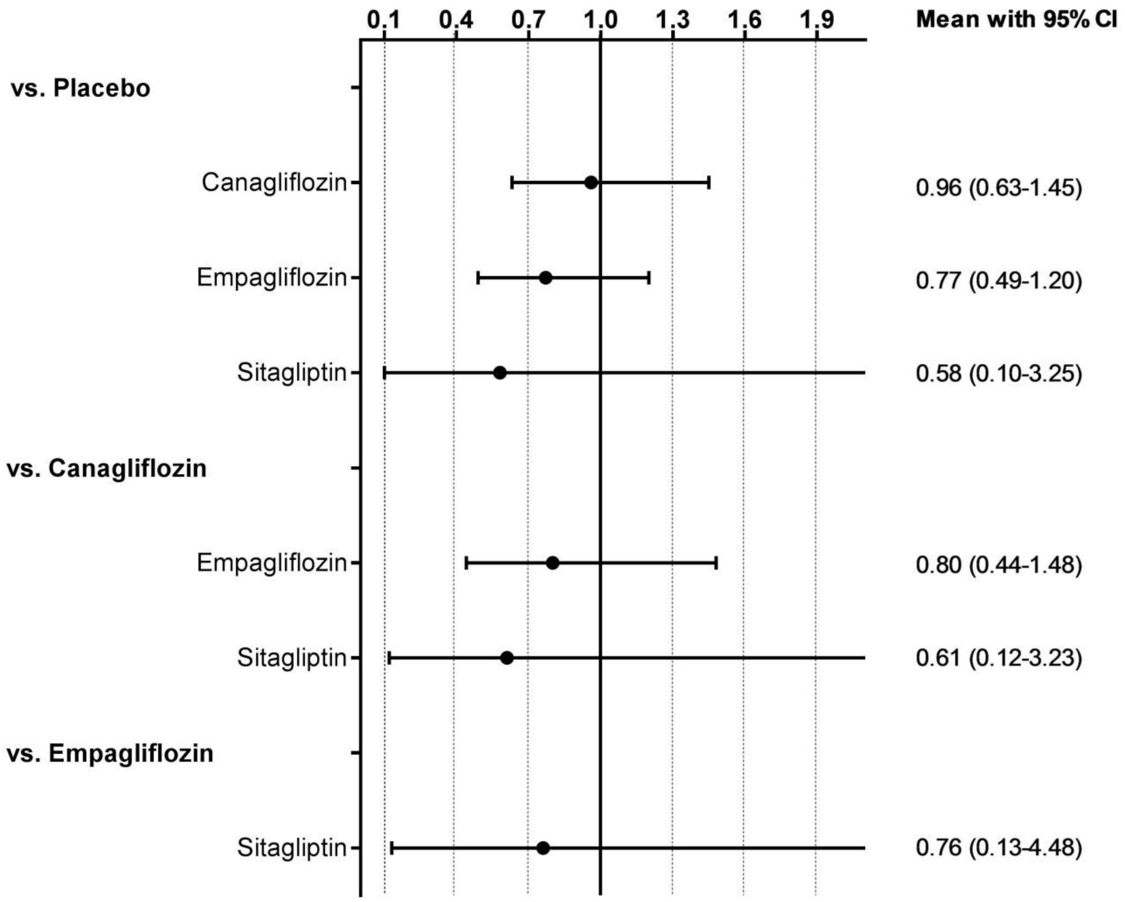

Legend: CV, cardiovascular; HF, heart failure; RF, renal function; T2D, type 2 diabetes mellitus

SUPPLEMENTARY TABLES

Online Table 1: Characteristics of SGLT2i included in analyses

Name of drug	Bioavailability	Protein binding	t_{max}	t½	C_{max}	SGLT2 selectivity over SGLT1
Canagliflozin	65% (300 mg dose)	99%	1-2 h	10.6 h (100 mg dose)<br>13.1 h (300 mg dose)	1,096 ng/ml (100 mg dose)<br>3,480 ng/ml (300 mg dose)	250 fold
Dapagliflozin	78%	91%	1-1.5 h	12.9 h	79.6 ng/ml (5 mg dose)<br>165.0 ng/ml (10 mg dose)	1,200 fold
Empagliflozin	78%	86.2%	1.5 h	13.2 h (10 mg dose)<br>13.3 h (20 mg)	259 ng/ml (10 mg dose)<br>687 ng/ml (20 mg dose)	2,500 fold

Ertugliflozin	70-90%	95%	0.5-1.5 h	11-17 h	268 ng/ml (15 mg dose)	2,000 fold
Licogliflozin	77%	n/a	0.75 h	17.2 h	1,480 ng/ml	35 fold
Sotagliflozin	>50%	>90%	3.0 h	13.2 h	64 ng/ml	20 fold

(52, 62-64)

C_{max}, maximum serum concentration that drug achieves in body after the drug has been and administrated; n/a, not available; SGLT, sodium-glucose cotransporter; t_{max}, time to achieve maximum plasma concentration; t½, biological half-life.

Online Table 2: Characteristics of HF patients included in SGLT2i trials

**Record**	**Trial ***	**Patients** ***(n)***	**Age** ***(years)***	**Female** ***(n, %)***	**LVEF** ***(%)***	**HbA1c** ***(%)***	**T2D** ***(n, %)***	**eGFR** ***(ml/min /1.73m²)***	**Background diabetes treatment**	**Background HF treatment**
Bhatt (8)	SOLOIST-WHF	1,222	70	412 (33.7)	35	7.1	1,222 (100)	49.7	any (52% metformin, 16% DPP4i, 3% GLP1-RA, 19% sulfonylurea, 36% insulin)	83% ACEI/ARB, 17% ARNI, 92% BB, 64% MRA, 95% diuretics
Butler (25), Fitchett (26, 27)	EMPA-REG-OUTCOME	706	63	211 (29.9)	n/a	8.1	706 (100)	68.7	any	87% ACEI/ARB, 79% BB, 24% MRA, 72% diuretics
Cannon (28), Cosentino (29)	VETIS CV	1,958	64	624 (31.8)	n/a	7.1	1,958 (100)	n/a	any	84% ACEI/ARB,

										79% BB, 19% MRA, 57% diuretics
Carbone (30)	CANA-HF	36	56 ± 7.8	8 (22.2)	29	8.3	36 (100)	79.2	any (56% metformin, 3% DPP4i, 3% GLP1-RA, 17% sulfonylurea, 47% insulin)	69% ACEI/ARB, 17% ARNI, 94% BB, 61% MRA, 86% diuretics
de Boer (31)	licogliflozin vs. empagliflozin vs. placebo	124 †	69	35 (28.2)	n/a	n/a	124 (100)	67	any (70% metformin, 18% DPP4i, 2% GLP1-RA, 2% alpha glucosidase inhibitor, 21% sulfonylurea, 49% insulin)	3% ACEI/ARB, 1% ARNI, 2% diuretics

Jensen (32)	EMPIRE-HF	190	64	28 (14.7)	29 (25-35)	5.8	33 (17.4)	74	any (9% metformin, 3% DPP4i, 3% GLP1-RA, 2% sulfonylurea, 4% insulin)	65% ACEI/ARB, 31% ARNI, 95% BB, 66% MRA, 64% diuretics
Kato (33)	DECLARE	1,987	64	669 (33.7)	n/a	8.2	1,987 (100)	84	any	86% ACEI/ARB, 81% BB, 19% MRA, 64% diuretics
Kosiborod (34) ‡	1. Moderate KD (56)<br>2. Add-on to sulfonylurea (57)<br>3. Add-on to insulin (58)<br>4. High CV risk (59)<br>5. High CV risk (60)	1. 19<br>2. 13<br>3. 18<br>4. 118<br>5. 152	64	119 (37.2)	n/a	8.2 ±0.9	320 (100)	70.3	1. any<br>2. glimepiride<br>3. insulin ± OAD<br>4. OAD ± insulin<br>5. OAD ± insulin	88% ACEI/ARB, 82% BB, 16% MRA, 63% diuretics

		∑ = 320								
Lee (35)	SUGAR-DM-HF	105	68.7 ± 11.1	28 (26.7)	32.5 ± 9.8	7.2 ± 1.5	82 (78.1)	67.3 ± 22.0	any (75% metformin, 36% DPP4i, 8% GLP1-RA, 41% sulfonylurea, 15% insulin)	61% ACEI/ARB, 34% ARNI, 91% BB, 60% MRA, 57% diuretics
McMurray (6, 36), Petrie (37)	DAPA-HF	4,744	66	1,091 (23)	31	6.5	2139 (45.1)	66	any (21% metformin, 7% DPP4i, 0.4% GLP1-RA, 9% sulfonylurea, 11% insulin)	84% ACEI/ARB, 11% ARNI, 96% BB, 71% MRA, 93% diuretics
Nassif (38)	DEFINE-HF	263	61	70 (26.6)	26	7.2	166 (63.1)	69.1	any (24% metformin, 8% DPP4i, 2% GLP1-RA, 13%	59% ACEI/ARB, 32% ARNI, 97% BB, 61%

									sulfonylurea, 33% insulin)	MRA, 86% diuretics
Packer (7), Anker (39)	EMPEROR-Reduced	3,730	67	893 (26.5)	27	n/a	1,856 (49.8)	62	any	70% ACEI/ARB, 19% ARNI, 95% BB, 71% MRA
Perkovic (40)	CREDENCE	652	n/a	n/a	n/a	n/a	652 (100)	n/a	any	n/a
Radholm (41)	CANVAS Program	1,461	63.8 ± 8.3	648 (44.4)	n/a	8.4 ± 1.0	1,461 (100)	73.0 ± 19.6	any (68% metformin, 8% DPP4i, 2% GLP1-RA, 45% sulfonylurea, 1% thiazolidinedione, 48% insulin)	86% ACEI/ARB, 70% BB, 60% diuretics

Santos-Gallego (42)	EMPA-TROPISM	84	62 ± 12.1	30 (36)	36 ± 8	5.8	0 (0)	82	none	42% ACEI/ARB, 43% ARNI, 88% BB, 33% MRA, 61% diuretics
Singh (43)	REFORM	56	67.1	19 (33.9)	45	7.7	56 (100)	72	any (55% metformin, 39% other OAD, 29% insulin)	89% ACEI/ARB, 82% BB, 41% MRA
Boehringer Ingelheim (44) §	EMPERIAL-Reduced	312	69.0 ± 10.2	80 (25.6)	n/a	n/a	n/a	n/a	any	any
Boehringer Ingelheim (45) §	EMPERIAL-Preserved	315	73.5 ± 8.8	136 (43.2)	n/a	n/a	n/a	n/a	any	any

ACEI, angiotensin converting enzyme inhibitor; ARB, angiotensin receptor blocker; ARNI, angiotensin receptor neprilysin inhibitor; BB, beta-blocker; CV, cardiovascular; DPP4i, dipeptidyl peptidase-4 inhibitor; eGFR, estimated glomerular filtration rate; GLP1-RA, glucagon-like peptide-1 receptor agonist; HF, heart failure; LVEF, left ventricular ejection fraction; MRA, mineralocorticoid receptor antagonist; n/a, not available; OAD, oral antidiabetic drug; SGLT2i, sodium-glucose cotransporter 2 inhibitor; T2D, type 2

diabetes mellitus.

* Acronym/ short title.

† Of the 124 patients randomized in the study, 80 were discontinued due to early study termination, with 44 patients completing the 12-weeks study.

‡ This record presents a HF subgroup meta-analysis from five randomized controlled trials. Patients with HF included in any of the five trials have been identified retrospectively and data have been pooled for joint analyses.

§ At the time of the literature search, results have not been published in a journal but were extracted from www.clinicaltrials.gov.

Online Table 3: Endpoint characteristics of trials analyzing the effects of SGLT2i in patients with HF

Record	Trial *	Hospitalization for HF		All-cause mortality		CV mortality		Worsening RF		Worsening HF or CV death		Worsening RF or CV death	
		Tx	Ctrl	Tx	Ctrl	Tx	Ctrl	Tx	Ctrl	Tx	Ctrl	Tx	Ctrl
Bhatt (8)	SOLOIST-WHF	194/608 †	297/614 †	65/608	76/614	51/608	58/614	n/a	n/a	245/608 †	355/614 †	n/a	n/a
Butler (25) Fitchett (26, 27)	EMPA-REG OUTCOME	48/462	30/244	56/462	35/244	38/462	27/244	21/458	14/241	75/462	49/244	56/462	37/241
Cannon (28), Cosentino (29)	VERTIS CV	69/ 1,286	55/672	n/a	n/a	n/a	n/a	n/a	n/a	164/ 1,286	99/672	n/a	n/a
Carbone (30)	CANA-HF	1/17	0/19	0/17	0/19	0/17	0/19	4/17	3/19	1/17	0/19	4/17	3/19
de Boer (31)	licogliflozin vs. empagliflozin vs.	n/a	n/a	1/61	0/30 1/33	1/61	0/30 0/33	n/a	n/a	n/a	n/a	n/a	n/a

	placebo												
Jensen (32)	EMPIRE-HF	1/95	0/95	0/95	0/95	0/95	0/95	n/a	n/a	1/95	0/95	n/a	n/a
Kato (33)	DECLARE	92/980	130/ 1,007	122/980	149/ 1,007	79/980	85/ 1,007	n/a	n/a	151/980	194/ 1,007	n/a	n/a
Kosiborod (34) ‡	1. Moderate KD (56) 2. Add-on to sulfonylurea (57) 3. Add-on to insulin (58) 4. High CV risk (59) 5. High CV risk (60)	1/171	7/149	0/171	1/149	n/a	n/a	n/a	n/a	n/a	n/a	n/a	n/a
Lee (35)	SUGAR-DM-HF	n/a	n/a	2/52	0/53	1/52	0/53	n/a	n/a	n/a	n/a	n/a	n/a
McMurray (6, 36), Petrie (37)	DAPA-HF	231/ 2,373	318/ 2,371	276/ 2,373	329/ 2,371	227/ 2,373	273/ 2,371	28/ 2,373	39/ 2,371	382/ 2,373	495/ 2,371	n/a	n/a
Nassif (38)	DEFINE-HF	10/131	8/132	1/131	1/132	1/131	1/132	1/131	1/132	n/a	n/a	n/a	n/a

Packer (7), Anker (98)	EMPEROR-Reduced	246/ 1,863	342/ 1,867	249/ 1,863	266/ 1,867	187/ 1,863	202/ 1,867	30/ 1,863	58/ 1,867	361/ 1,863	462/ 1,867	n/a	n/a
Perkovic (40)	CREDENCE	n/a	n/a	n/a	n/a	n/a	n/a	n/a	n/a	n/a	n/a	52/329	53/323
Radholm (41)	CANVAS	35/803	57/658	74/803	79/658	60/803	64/658	17/803	22/658	88/803	115/658	n/a	n/a
Santos-Gallego (42)	EMPA-TROPISM	0/42	2/42	0/42	1/42	0/42	1/42	n/a	n/a	n/a	n/a	n/a	n/a
Singh (43)	REFORM	1/28	1/28	1/28	4/28	0/28	3/28	n/a	n/a	n/a	n/a	n/a	n/a
Boehringer Ingelheim (44) §	EMPERIAL-Reduced	n/a	n/a	3/155	3/156	n/a	n/a	n/a	n/a	n/a	n/a	n/a	n/a
Boehringer Ingelheim (45) §	EMPERIAL-Preserved	n/a	n/a	1/157	0/158	n/a	n/a	n/a	n/a	n/a	n/a	n/a	n/a

Numbers indicate number of events/ number of patients.

CV, cardiovascular; Ctrl, control; HF, heart failure; n/a, not available; RF, renal function; SGLT2i, sodium-glucose cotransporter-2 inhibitor; T2D, type 2 diabetes mellitus; Tx, treatment.

* Acronym/ short title.

† Numbers include urgent HF visits.

‡ This record presents a HF subgroup meta-analysis from five randomized controlled trials. Patients with HF included in any of the five trials have been identified retrospectively and data have been pooled for joint analyses.

§ At the time of the literature search, results have not been published in a journal but were extracted from www.clinicaltrials.gov.
